# An Allosteric Mechanism for Switching between Parallel Tracks in Mammalian Sulfur Metabolism

**DOI:** 10.1371/journal.pcbi.1000076

**Published:** 2008-05-02

**Authors:** Tatyana K. Korendyaseva, Denis N. Kuvatov, Vladimir A. Volkov, Michael V. Martinov, Victor M. Vitvitsky, Ruma Banerjee, Fazoil I. Ataullakhanov

**Affiliations:** 1 National Research Center for Hematology, RAMS, Moscow, Russia; 2 Biological Chemistry Department, University of Michigan, Ann Arbor, Michigan, United States of America; 3 Department of Physics, Moscow State University, Moscow, Russia; 4 Center for Theoretical Problems of Physicochemical Pharmacology, RAS, Moscow, Russia; Boston University, United States of America

## Abstract

Methionine (Met) is an essential amino acid that is needed for the synthesis of S-adenosylmethionine (AdoMet), the major biological methylating agent. Methionine used for AdoMet synthesis can be replenished via remethylation of homocysteine. Alternatively, homocysteine can be converted to cysteine via the transsulfuration pathway. Aberrations in methionine metabolism are associated with a number of complex diseases, including cancer, anemia, and neurodegenerative diseases. The concentration of methionine in blood and in organs is tightly regulated. Liver plays a key role in buffering blood methionine levels, and an interesting feature of its metabolism is that parallel tracks exist for the synthesis and utilization of AdoMet. To elucidate the molecular mechanism that controls metabolic fluxes in liver methionine metabolism, we have studied the dependencies of AdoMet concentration and methionine consumption rate on methionine concentration in native murine hepatocytes at physiologically relevant concentrations (40–400 µM). We find that both [AdoMet] and methionine consumption rates do not change gradually with an increase in [Met] but rise sharply (∼10-fold) in the narrow Met interval from 50 to 100 µM. Analysis of our experimental data using a mathematical model reveals that the sharp increase in [AdoMet] and the methionine consumption rate observed within the trigger zone are associated with metabolic switching from methionine conservation to disposal, regulated allosterically by switching between parallel pathways. This regulatory switch is triggered by [Met] and provides a mechanism for stabilization of methionine levels in blood over wide variations in dietary methionine intake.

## Author Summary

Methionine is an essential amino acid that is highly toxic at elevated levels, and the liver is primarily responsible for buffering its concentration in circulation. Intracellularly, methionine is needed for the synthesis of S-adenosylmethionine (AdoMet), the major biological methylating agent. Methionine used for AdoMet synthesis can be replenished via remethylation of homocysteine. Alternatively, homocysteine can be converted to cysteine via the transsulfuration pathway. A specific feature of liver methionine metabolism is the existence of twin pathways for AdoMet synthesis and degradation. In this study, we analyzed the dependence of methionine metabolism on methionine concentration in liver cells using a combined experimental and theoretical approach. We find a sharp transition in rat hepatocyte metabolism from methionine conservation to a disposal mode with an increase in methionine concentration above its physiological range. Mathematical modeling reveals that this transition is afforded by an allosteric mechanism for switching between parallel metabolic pathways. This study demonstrates a novel mechanism of trigger behavior in biological systems by which the substrate for the metabolic network switches metabolic flux between parallel tracks for sustaining two different metabolic modes.

## Introduction

**Figure 1:**
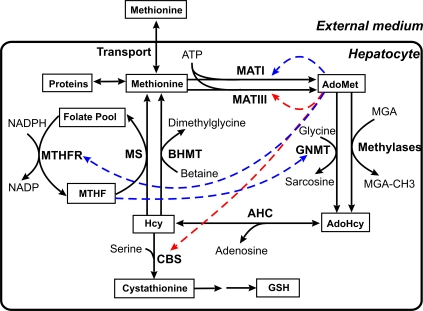
Scheme for liver methionine metabolism. The solid arrows show enzymatic reactions. Dashed arrows indicate the allosteric regulatory influence of metabolites to enzymes. Red and blue colors indicate activation and inhibition respectively. Besides its role in protein synthesis, methionine is a substrate for the synthesis of S-adenosylmethionine (AdoMet), which is a ubiquitous methyl donor for numerous methyltransferases and is a precursor for polyamine synthesis. A product of the methyltransferase reactions is S-adenosylhomocysteine (AdoHcy), which in turn, is cleaved to adenosine and homocysteine in a reaction catalyzed by S-adenosylhomocysteinase (AHC). Homocysteine can be remethylated to methionine using the methyl group of methyl tetrahydrofolate (MTHF), or betaine, in reactions catalyzed by methionine synthase (MS) and betaine homocysteine methyltransferase (BHMT), respectively. In many tissues, homocysteine is also metabolized via the transsulfuration pathway to generate cysteine, the limiting substrate for synthesis of glutathione (GSH), a major intracellular antioxidant. Enzyme abbreviations: AHC: adenosylhomocysteinase; BHMT: betaine homocysteine S-methyltransferase; CBS: cystathionine β-synthase; GNMT: glycine-N-methyltransferase; MATI, III: methionine adenosyltransferase I, III; MS: methionine synthase; MTHFR: methylenetetrahydrofolate reductase. Metabolite abbreviations: AdoHcy: S-adenosylhomocysteine; AdoMet: S-adenosylmethionine; Hcy: homocysteine; MGA: methyl group acceptor; MTHF: 5-methyltetrahydrofolate; GSH: glutathione.

Methionine, an essential amino acid, plays a significant role in intracellular one-carbon and sulfur metabolism consequently linking antioxidant and methylation homeostasis (Figure 1). Since methionine is involved in many intracellular processes its levels in blood and in different organs need to be tightly regulated. In fact, methionine levels in blood and liver do not change over a several-fold variation in dietary methionine intake [1]–[3]. Aberrations in methionine, and therefore in methylation and antioxidant metabolism, are associated with a number of complex diseases, including cancer, anemia, neurodegenerative diseases, and developmental abnormalities [4],[5]. Hence, elucidating the multiple switches that regulate methionine metabolism is key to understanding their dysregulation. Such information may also permit intervention that would reverse disease states.

Liver is considered to be the main organ that removes excess methionine from the organism and regulates the methionine level in blood. The metabolism of methionine in liver is more complex than in other organs; it is characterized by a unique, liver-specific enzyme profile (Figure 1), which includes the methionine adenosyltransferase I (MATI) and methionine adenosyltransferase III (MATIII) isoforms of methionine adenosyltransferase. Methionine adenosyltransferase II (MATII) is present only in extrahepatic tissues. The enzymatic reaction of the MATIII isoform exhibits a rate dependence on AdoMet concentration that is the converse of MATI and MATII. Thus, while MATI, like MATII, is inhibited by AdoMet, MATIII is activated at high concentrations of AdoMet [6],[7]. Liver also possesses glycine-N-methyltransferase (GNMT), betaine homocysteine S-methyltransferase (BHMT), and cystathionine β-synthase (CBS) at levels that are much higher than those found in other organs and tissues [8]. Thus, an interesting feature of liver methionine metabolism is that there are parallel tracks for the synthesis and utilization of AdoMet, and at the homocysteine junction, methionine can either be recycled or committed to the synthesis of cysteine, which can then be either utilized biosynthetically or catabolized (Figure 1).

Despite the large number of studies on methionine metabolism in liver [8] its regulation as well as the kinetics of the methionine disposal, are not well understood. Furthermore, the dependence of liver methionine metabolism on fluctuating methionine concentration ([Met]) within a physiologically relevant range has not been investigated. To elucidate the molecular mechanism that controls metabolic fluxes via parallel tracks in liver methionine metabolism, we had developed a simple mathematical model [9], which revealed the possibility of a threshold dependence of liver methionine metabolism on [Met]. This simple qualitative model predicted the existence of two modes in liver methionine metabolism characterized by low metabolic rates and metabolite levels at methionine concentrations equal to or below its normal physiological value and by high metabolic rates and metabolite concentrations at methionine concentrations above its physiological value. The model predicted that under a specific set of conditions, methionine metabolism switches sharply from one mode to another when [Met] slightly exceeds its physiological value. The switch from the “low” to “high” mode is associated with a sharp increase in steady-state AdoMet concentrations. It was presumed that this metabolic switch enables conservation at low methionine levels and disposal at high methionine levels, and is associated with a redistribution of metabolic flux between remethylation and transsulfuration. However, a detailed analysis of the metabolic switch could not be undertaken with the simple model because it did not include descriptions of remethylation and transsulfuration fluxes in an explicit form. Moreover, the simple model had significant limitations such as the loss of stability at methionine concentrations exceeding the normal physiological value by ∼10% and unrealistically slow kinetics for transitional processes [9], raising questions about its utility for experimental verification at the high end of physiologically relevant methionine concentrations and on a real time scale.

In this study, we have analyzed the experimental dependencies of [AdoMet] and methionine consumption rate on [Met] in native murine hepatocytes within a physiologically relevant concentration range (40 to 400 µM). We have found that both [AdoMet] and methionine consumption rate do not respond gradually with increasing [Met] but rise sharply, increasing ∼10-fold within the narrow [Met] concentration interval from 50 to 100 µM in accordance with the predictions of our preliminary mathematical modeling of this pathway [9]. We used our experimental results for construction and quantitative verification of the extended mathematical model of liver methionine metabolism, which included a detailed description of the kinetics of all the relevant enzymes, a simplified description of folate metabolism and a new rate equation for the MATIII reaction. The new rate equation for the MATIII reaction was developed to describe the experimental data at methionine concentrations above its normal physiological value. Analysis of the model reveals that the sharp increase in [AdoMet] and methionine consumption rate observed in hepatocytes at increasing [Met] are associated with a sharp transition in methionine metabolism from a conservation to a disposal mode (i.e. from remethylation to transsulfuration). The transition is controlled by allosteric regulation of enzymes that results in switching metabolic flux between parallel pathways for AdoMet synthesis and utilization. This metabolic switch is triggered by methionine and provides a mechanism for stabilization of methionine levels in blood over wide variations in dietary methionine intake.

Our study reveals an excellent correspondence between the experimental and predicted behavior of liver cells in response to variations in [Met] and demonstrates the existence of a novel allosteric mechanism for switching metabolic fluxes between parallel pathways.

## Results

### Effect of Initial Concentration of Methionine on the Intracellular Concentrations of AdoMet, AdoHcy, and Methionine Consumption Rate in Murine Hepatocytes

**Figure 2:**
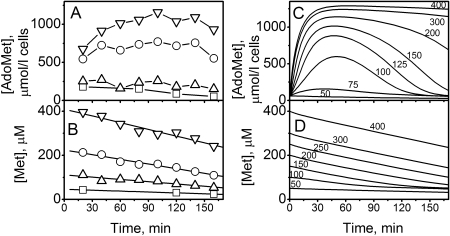
Kinetics of [AdoMet] and [Met] in a suspension of murine hepatocytes incubated at different initial [Met]. (A,B) Data from a representative experiment obtained at a cell density of 1·10^6^ cells/ml and initial [Met] of □-40, ▵-120, ○-240, and ▿-400 µmol/l respectively. (C,D) Model simulation, calculated using a complete system of equations (Text S1 and Equation S1) at *w_med_/w_hep_* = 99 (1% cell suspension) and V^influx^ = 0. Initially, all model variables except [Met] were set to the normal physiological steady-state values (Table S3), and calculations were started at different initial [Met] values as indicated above each line.

In a suspension of murine hepatocytes incubated with 40 µM methionine, the intracellular concentration of AdoMet was quite stable over 3 h. The average value of [AdoMet] obtained under these conditions in 24 independent experiments was 79±37 µmol/l cells (mean±SD). At higher methionine concentrations, the intracellular [AdoMet] increased within 20–30 min and then reached a plateau within ∼1 h, representing a new steady-state level, which did not change further during the following 1–2 h (Figure 2A). As the initial [Met] in the suspension was increased, the steady-state AdoMet level increased monotonically. However, the major increase in [AdoMet] was observed at an initial [Met] above 200 µM. When the initial [Met] was 200–240 µM or 400 µM, the intracellular AdoMet level reached values of 590±180 (n = 9) and 930±350 µmol/l cells (n = 12) respectively, exceeding the concentration obtained at 40 µM methionine by 11- to 15-fold, on average.

Under control conditions, i.e., at 40 µM methionine, the average intracellular concentration of S-adenosylhomocysteine (AdoHcy), obtained in 7 independent experiments was 7.1±7.0 µmol/l cells. The concentration of AdoHcy also increased with the increase in initial [Met] in the cell suspension, reaching a value of 76±21 µmol/l cells at an initial [Met] of 400 µM. This exceeded the concentration obtained at 40 µM methionine, on average, by 10-fold (not shown).

The concentration of methionine in the incubation medium decreased with time, due to methionine consumption by hepatocytes (Figure 2B). The dependence of [Met] on time was approximated by linear kinetics, and the slope of the corresponding line normalized to cell volume was taken as the rate of methionine consumption by hepatocytes. The rate of methionine consumption increased with an increase in the initial [Met]. At 40 µM methionine, the average rate of methionine consumption, obtained in 15 independent experiments, was 0.86±0.40 mmol/h·l cells. The rate of methionine consumption by hepatocytes increased to 6.0±2.4 mmol/h·l cells at an initial [Met] of 400 µM (n = 12), exceeding its value at 40 µM methionine by 8.1±3.6-fold. Our mathematical model, which is described in the Methods section and in Supporting Text S1 and S2, provides a satisfactory description of the [AdoMet] and [Met] kinetics observed experimentally in hepatocytes in response to changes in [Met] (Figure 2C, D).

### Dependence of Steady-State AdoMet and AdoHcy Concentrations in Murine Hepatocytes on Methionine Concentration

**Figure 3:**
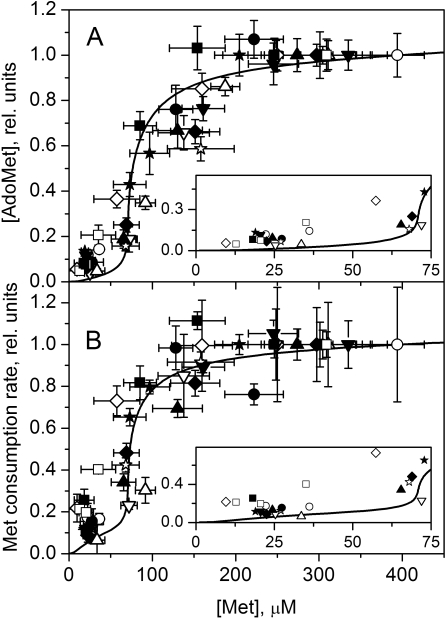
The methionine concentration dependence of steady-state values of (A) [AdoMet] and (B) the rate of methionine consumption in mouse hepatocytes. Symbols indicate experimental data obtained in 12 independent experiments, with each symbol representing a single experiment. Solid lines show the results of mathematical modeling calculated using the parameter values indicated in Tables S1 and S2. Each experimental data point for [AdoMet] shows the mean±SD of samples collected at least in triplicate in the steady-state phase of AdoMet kinetics. Each experimental data point for methionine consumption rate shows the result of a linear fit (mean±SD) of methionine kinetics, obtained with samples collected between 20 and 160 min of cell incubation. Horizontal error bars show the methionine concentration intervals corresponding to the steady-state AdoMet concentrations. In each experiment, values for [AdoMet] and methionine consumption rate were normalized to values obtained at an initial methionine concentration of 400 µM. The theoretical curves were normalized to values obtained at 400 µM methionine. Insets in both (A) and (B) show the experimental (symbols) and simulated (line) data in greater detail at low [Met]. Error bars were removed for clarity.

To assess the experimental dependence of steady-state AdoMet concentration on methionine, we plotted the average intracellular [AdoMet] measured between 60 and 160 min of incubation versus the average [Met] measured during the same time interval. The AdoMet concentration obtained in each experiment was normalized to the maximum AdoMet levels obtained at an initial [Met] of 400 µM with the same batch of cells. Data from 12 independent experiments are presented in Figure 3A. As can be seen, a distinctive feature of the dependence of [AdoMet] on [Met] in mouse hepatocytes is the sharp increase in [AdoMet] within a narrow range of [Met] between 50 and 100 µM. Below and above this trigger zone, the steady-state [AdoMet] increases only slightly with an increase in [Met]. The relatively low slope of the experimentally observed dependence of [AdoMet] on [Met] at low methionine concentrations (Figure 3A, *inset*) is incompatible with a simple hyperbolic dependence.

**Figure 4:**
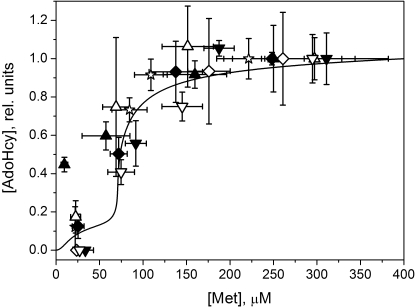
The dependence of steady-state AdoHcy concentration on methionine in mouse hepatocytes. Symbols indicate experimental data obtained in 7 independent experiments. Data obtained in the same experiment are shown by the same symbols used in Figures 3 and 4. The solid line shows the result of mathematical modeling calculated using the parameter values indicated in Tables S1 and S2. Each experimental data point for [AdoHcy] is represented as the mean±SD of at least 3 samples. Horizontal error bars show methionine concentration intervals corresponding to the steady-state AdoMet concentrations. In each experiment, values for [AdoHcy] were normalized to value obtained at an initial methionine concentration of 400 µM. Similarly, theoretical data were normalized to the value obtained at 400 µM methionine.

The quantitative mathematical model developed in the present work exhibits a sharp increase in [AdoMet] within a narrow concentration range of methionine above its physiological level (Figure 3A), in good agreement with the experimental results. We have similarly examined the dependence of steady-state AdoHcy concentration on methionine (Figure 4). This dependence is similar to the dependence of [AdoMet] on [Met] (Figure 3A) and is well described by the model (Figure 4).

### Dependence of the Methionine Consumption Rate on Methionine Concentration in Murine Hepatocytes

The experimentally derived rate for methionine consumption in hepatocytes was plotted versus the average [Met] measured between 60 and 160 min, as described above for AdoMet (Figure 3B). The values obtained for the methionine consumption rate in each experiment were normalized to the rate obtained with the same batch of cells at an initial [Met] of 400 µM. As seen for the behavior of the intracellular AdoMet concentration, the methionine consumption rate increased several-fold in a relatively narrow concentration interval of methionine and then did not change so significantly. The experimental data are in good agreement with the prediction of the mathematical model (Figure 3B). The relatively low slope at low [Met] is inconsistent with the methionine consumption rate being a simple hyperbolic function of [Met] (Figure 3B, *inset*).

### Metabolic Switch Between Transmethylation and Transsulfuration (Methionine Conservation and Disposal)

**Figure 5:**
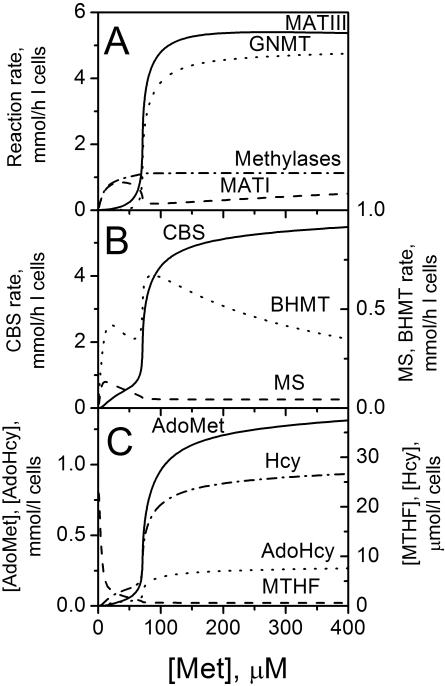
The dependence of (A, B) steady-state reaction rates and (C) metabolite concentrations in methionine metabolism on [Met] as calculated by the model. The enzyme and metabolite abbreviations are denoted by the curves corresponding to their behavior. All parameter values used for the calculations are given in Tables S1 and S2.

The extended model supports analysis of specific reaction rates and metabolites in the sharp transition between low and high [AdoMet] and methionine consumption rate within a narrow [Met] range. The model reveals that at physiological [Met] (∼50 µM), almost all the AdoMet is produced by the MATI isoform and consumed by cellular methylases, thereby meeting the demands for methylation reactions (Figure 5A). Under these conditions, the futile metabolic flux through GNMT is very low because [AdoMet] is low and high levels of 5-methyltetrahydrofolate (MTHF) inhibit GNMT (Figure 1, Figure 5C). Note that the steady-state rate of AdoMet production (*V^MATI^*+*V^MATIII^*) (for complete definition of terms, see Methods) is not equal to the net rate of methionine consumption (*V^MATI^*+*V^MATIII^*−*V^MS^*−*V^BHMT^*) because a significant amount of AdoMet is produced due to recirculation of metabolites via methionine synthase (MS) and betaine homocysteine S-methyltransferase (BHMT) (Figure 5). A small increase in [Met] above its normal value leads to a moderate increase in [AdoMet]. Due to the opposing influence of AdoMet on MATI and MATIII isoforms (Figure 1), the rate of MATI decreases, while the rate of MATIII increases and the total rate of AdoMet production increases slightly. At a threshold value for methionine (∼70 µM in the model) the rate of the MATIII reaction exceeds the rate of the MATI reaction and the rate of AdoMet production exceeds the total activity of cellular methylases. This leads to a rapid and auto-accelerated accumulation of AdoMet and an increase in the rate of the MATIII reaction due to positive feedback regulation of MATIII by AdoMet, which cannot be compensated by a further decrease in the MATI reaction rate. Also, increased AdoMet levels inhibit methylenetetrahydrofolate reductase (MTHFR) that leads to a significant decrease in [MTHF]. Consequently, GNMT is strongly activated due to its sigmoidal dependence on [AdoMet] and decreased inhibition by MTHF under these conditions (Figure 1, Figure 5). The sharp activation of GNMT results in consumption of excess AdoMet produced by activated MATIII providing a new steady state of methionine metabolism characterized by high [AdoMet] and a high metabolic rate. This regulation provides the metabolic switch in methionine metabolism from the “low” to the “high” metabolic mode. Thus, above the threshold concentration for methionine, the rate of AdoMet synthesis increases several-fold, due mainly to the activity of MATIII. The contribution of MATI to total AdoMet synthesis decreases to <10% and excess AdoMet is metabolized via GNMT. Below the threshold [Met], a large fraction of the methionine used in AdoMet synthesis is regenerated via transmethylation reactions catalyzed by MS and BHMT, and the net rate of methionine consumption is low (Figure 5, Figure 3B, theoretical curve). Above the threshold, the net rate of methionine consumption increases dramatically (Figure 3B, theoretical curve), and methionine is primarily catabolized via the transsulfuration pathway (Figure 5B). The increased AdoMet concentration inhibits MTHFR that leads to a sharp decrease in the MS substrate, MTHF. This in turn, leads to a decrease in the MS reaction rate despite the increased level of the other substrate, homocysteine (Hcy). Meanwhile, the committing enzyme in the transsulfuration pathway, CBS, is activated due to an increase in the concentration of its substrate, homocysteine and its allosteric activator, AdoMet. These results are in good agreement with the published experimental data demonstrating the decrease in the fraction of methionine produced via remethylation and activation of methionine catabolism via the transsulfuration pathway at high [Met] in liver or hepatocytes [2],[10],[11].

### Mechanism of Stabilization of Methionine Concentration

**Figure 6:**
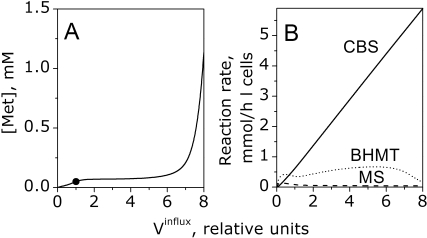
The influence of different methionine influx rates on the steady-state values of (A) [Met] and (B) CBS, MS, and BHMT reaction rates calculated by the model. The influx was normalized to a value of 0.76 mmol/h·l cells, which provides normal physiological steady-state [Met] value of 50 µM (indicated by the circle) in the model. All parameter values used for the calculations are given in Tables S1 and S2.

The metabolic switch between methionine conservation and disposal buffers methionine levels in liver and provides a mechanism for stabilizing liver and blood methionine concentrations over a wide range of dietary methionine intake. Indeed, rapid normalization of blood and liver methionine is observed after food consumption [12] or methionine injection [1],[13]. Blood and liver methionine levels do not vary significantly over a several-fold range in dietary methionine intake [1]–[3]. To demonstrate methionine stabilization in the extended model, we analyzed the dependence of steady-state methionine metabolism on the rate of methionine influx. Figure 6A shows the computed dependence of the steady-state [Met] on the normalized rate of methionine influx into hepatocytes. As one can see, [Met] is stabilized over a ∼6-fold increase in the rate of methionine influx. The rate of the CBS reaction increases proportionally with the increase in the rate of methionine influx while the rate of transmethylation, contributed collectively by MS and BHMT, does not change significantly decreasing only at very high values of methionine influx (Figure 6B). This indicates that stabilization of [Met] is provided via catabolism by adjusting the flux through the transsulfuration pathway.

## Discussion

The methionine metabolic pathway represents a useful paradigm for studying gene-nutrient interactions since it is richly dependent on the input of dietary factors (viz. amino acids and B-vitamins). Moreover, several genetic polymorphisms have been described in the pathway's enzymes that are correlated with risk for various complex diseases [14]–[17]. An integrated understanding of the regulation of methionine metabolism is important because of its critical role in modulating the two major homeostatic systems governing methylation and antioxidant metabolism, which are often dysregulated in complex diseases. Attaining this understanding is, however, a challenge because methionine metabolism is complex; the pathway has branches and cycles in addition to parallel fluxes at several steps of intermediate transformation. Further, regulation of the pathway enzymes by intermediates increases the complexity of the system. Mathematical modeling is a powerful tool for studying the mechanisms of regulation in complex metabolic systems and for analyzing the behavior of a system under different natural and experimentally induced conditions. Previously, simple mathematical models of liver methionine metabolism have been developed and used to analyze and predict the response of the system to changes in [Met] and other variables [9],[18],[19].

The most striking prediction of our previously published simple mathematical model describing methionine metabolism is that a substrate (methionine) can induce the sharp transition between two modes in liver methionine metabolism characterized by low metabolic rates and metabolite levels at methionine concentrations equal to or below its normal physiological value and by high metabolic rates and metabolite concentrations at methionine concentrations above its physiological value [9]. The model predicted that this transition is triggered within a narrow methionine concentration range. In addition to the limitations of the simple model discussed in the introduction, we could not verify or adjust the model parameters to obtain a quantitative description of methionine metabolism because of the scant information available on the dependence of metabolic fluxes on [Met] in liver or in hepatocytes. Even the rate of methionine consumption under normal physiological conditions was unknown.

To address these gaps, we experimentally determined the responsiveness of methionine metabolism in murine hepatocytes to varying concentrations of methionine. Our study revealed a sharp dependence of [AdoMet] and the rate of methionine consumption in hepatocytes on [Met] within a narrow range of extracellular [Met] that lies just above the physiological concentration of circulating methionine (∼50 µM). These data supported the development of an extended quantitative model of liver methionine metabolism, which also includes rate equations for MS, BHMT and CBS, and a simplified treatment of folate metabolism. Importantly, without the link between methionine and folate metabolism the extended model failed to provide a realistic description of the GNMT reaction rate, confirming the experimentally established regulation of GNMT by the folate derivative, MTHF. Enzymatic reaction rates were described using mechanism-based equations and the kinetics parameters were adjusted from ranges reported in the literature and our own experimental data. In addition, a new equation had to be developed to describe the MATIII reaction, including both activation and inhibition of the enzyme at high [AdoMet], in order to describe the experimental data at [Met] above its normal physiological value. A significant attribute of the extended model is that it provides a good quantitative description of our experimental results, including steady-state values and transitional processes over a wide range of [Met].

The extended model was used to analyze two types of experiments. To assess the response of hepatocytes ex vivo, to the parameter, i.e., varying [Met], the behavior of pathway metabolites and reaction rates were analyzed. To describe in vivo conditions, [Met] was considered as a model variable and the rate of methionine intake was considered as an independent parameter. A good agreement was observed between the simulated and observed ex vivo experimental data. Our experimental results demonstrate that a sharp increase in [AdoMet] in hepatocytes is accompanied by a several-fold increase in the methionine consumption rate (Figure 3). This behavior is reversible and liver metabolism switches back to a conservation mode when the [Met] decreases below the threshold level. Our analysis of the in vivo response to varying intake of methionine reveals that the metabolic switch between methionine conservation and disposal provides a mechanism for stabilizing liver and blood methionine concentrations over a wide range of dietary methionine intake. This mechanism is associated with the regulation of the CBS reaction rate (Figure 6) in addition to regulation by MATI/III and GNMT. Homeostasis requires that the steady-state transsulfuration flux must be equal to or less than the net rate of methionine consumption. The normal rate of methionine consumption observed in murine hepatocytes, was ∼1 mmol/h·l cells, which represents ∼1.3% of murine liver CBS activity under maximal velocity conditions [20]. In the model, under physiological conditions, the rate of the CBS reaction is ∼0.6% of CBS activity (Figure 5B and Table S1). Thus, our experimental and theoretical data reveal that under normal physiological conditions, liver CBS works at a very small fraction of its maximal capacity, and that it can be activated several fold at high [Met] to increase the volume of sulfur flowing through the transsulfuration pathway.

**Figure 7:**
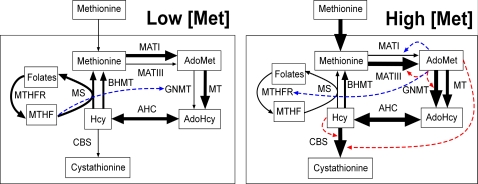
Scheme showing switching between parallel metabolic tracks triggered by the concentration of methionine. The width of the solid arrows is qualitatively proportional to the metabolic flux through each step. MT refers to functional methylases (methyltransferases). The dashed arrows indicate the influence of substrate and allosteric regulation, which determine flux in the “low” and “high” metabolic modes. The red and blue colors indicate activation and inhibition, respectively.

Thus, liver methionine metabolism exhibits trigger behavior that results from allosterically regulated switching between two sets of enzymes that catalyze parallel metabolic fluxes. In the methionine conservation mode, the metabolic flux is determined by one set of enzymes, which includes MATI and functional methylases, while in the methionine disposal mode, the metabolic flux is determined by a second set of enzymes that includes MATIII and GNMT (Figure 7). To our knowledge, such a mechanism of trigger behavior that causes metabolic flux to switch between parallel pathways has not been reported previously in other metabolic systems.

A sharp transition from one steady state to another in the background of a monotonic change in one parameter (or stimulus) is observed in many biological processes including ontogenesis, cell cycle and signal transduction. An ideal mechanism for trigger behavior is provided in a system that exhibits bistability, i.e., when an area of instability separates two stable steady states. In this case, the system can jump from one steady-state to the other, and stable intermediate states do not exist in the pathway. There are a number of theoretical and experimental reports of bistability in different biological processes [21]–[25]. In fact, within a fairly wide range of parameters, both the simple and extended models predict the existence of bistability in the system, leading to the trigger behavior in metabolite concentrations and metabolic fluxes, including [AdoMet] that jumps from its physiological concentration to significantly higher levels when the [Met] crosses a threshold value. This bistability results in a hypersensitivity of methionine metabolism to changes in methionine levels within a narrow concentration interval. The theoretical mechanism of bistability, which can be realized in liver methionine metabolism, is described in Text S3 and Figures S3, S4, and S5.

**Figure 8:**
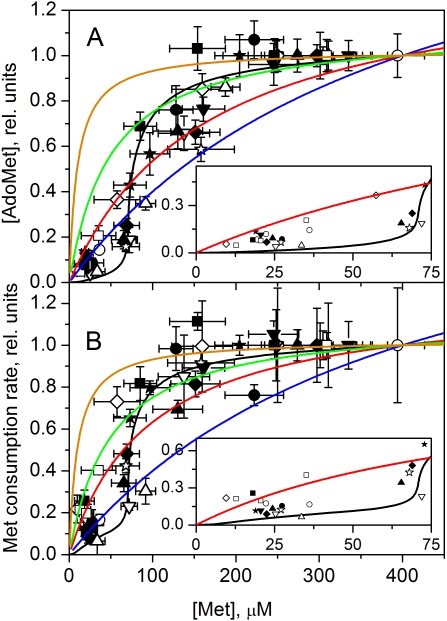
Comparison between model-based and Michaelis-Menten hyperbolic (*Y* = *X*/(*X*+*Km*) fits to the experimental data. The data points and fits in black are the same as shown in Figure 3. The red curves show the hyperbolic best fit using values for *Km* of 163 µM (A), and 96 µM (B). The other curves show hyperbolic traces calculated at *Km* values of 400 µM (blue), 50 µM (green), and 10 µM (orange). For each curve the value obtained at 400 µM [Met] was taken as 1.0.

However, bistability is not absolutely essential for effective regulation of liver methionine metabolism. Our model shows that even sharp monotonic dependence of methionine metabolism on methionine in a narrow range of [Met] produces metabolic switching between methionine conservation and disposal modes, providing a mechanism for effective stabilization of methionine levels. The results demonstrate that both [AdoMet] and the rate of methionine consumption in hepatocytes increase slowly with an increase in methionine level at low [Met] (0–50 µM) and then increase sharply in a narrow concentration range of 50–100 µM (Figure 3) in accordance with model predictions. While the experimental data cannot distinguish between a jump versus a monotonic increase in [AdoMet] and the rate of methionine consumption with an increase in [Met] they are not well described by a simple hyperbolic behavior (Figure 8). Formal approximations of the initial part of our experimental points at [Met] from 0 to 100 µM with the simple power function: Y = A+B*X^n^, provide the best fit at n = 2.30 for AdoMet and at n = 1.43 for methionine consumption rate. This is consistent with a sharp, non hyperbolic increase in [AdoMet] and methionine consumption rate in a narrow interval of [Met], confirming our model predictions. Similar power function approximation of regular Michaelis-Menten hyperbola Y = X/(100+X) gives n = 0.64.

The sharp change in methionine metabolism observed in hepatocytes at increasing methionine concentration is in striking contrast to the relatively weak dependence we have previously observed in hepatoma cells [18]. A notable difference between primary versus transformed hepatocytes is that the latter express the MATII isoenzyme instead of MATI/MATIII; they also lack GNMT [26]–[28]. Thus, our theoretical and experimental results demonstrate that the presence of the MATI/MATIII and GNMT is crucial for normal regulation of liver methionine metabolism and for achieving a normal balance between methionine conservation and disposal, thereby buffering methionine levels.

We note that our mathematical model describing methionine metabolism is still relatively simple. For instance, it does not include factors that can potentially affect liver and blood methionine levels, such as the modulation of enzyme levels by dietary factors [8] and methionine turnover in organs other than liver. Nevertheless, the predictive power of the model in revealing an unexpected mode of substrate-triggered regulation is validated by the results reported in this study, affirming the utility of a combined theoretical and experimental approach for the study of metabolic regulation.

## Materials and Methods

### Mathematical Model

Our mathematical model is a system of ordinary differential equations that describe the kinetics of extracellular and intracellular concentrations of methionine as well as intracellular concentrations of other intermediates of methionine metabolism (Figure 1).
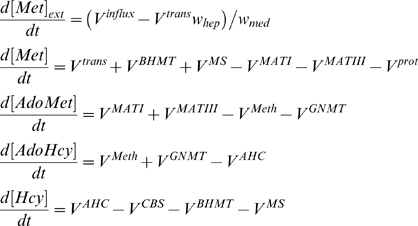
(1)


Here [Met]_ext_ and [Met] are the extracellular and intracellular methionine concentrations, *V^influx^* is the rate of methionine influx into the system, *V^trans^* is the rate of methionine transport into hepatocytes, *w_hep_* and *w_med_* are total volumes of hepatocytes and external medium, respectively, *V^prot^* is the net rate of methionine consumption in protein turnover, *V^MATI^*, *V^MATIII^*, *V^GNMT^*, *V^Meth^*, *V^AHC^*, *V^CBS^*, *V^MS^*, *V^BHMT^* are the rates of reactions, catalyzed by MATI, MATIII, GNMT, functional methylases, adenosylhomocysteinase (AHC), CBS, MS, and BHMT respectively.

The reversible AHC-catalyzed reaction is assumed to be at equilibrium because the activity of AHC is much higher than the activity of other enzymes in the metabolic system (see Table S1). The rate of methionine consumption in protein turnover and the total rate of functional methylases are described by simplified equations. Equations for rates of other enzymatic reactions are based on known kinetics mechanisms for the respective enzymes. We developed a new equation for the MATIII reaction in order to describe the experimental data at [Met] above its normal physiological value. All equations for the enzymatic reaction rates are presented in Text S1. The development of the equation for MATIII is described in Text S2 and Figures S1 and S2.

We do not take into account AdoMet consumption for polyamine synthesis because its rate is about 1% of flux in methionine metabolism [29] in normal liver.

The distribution of methionine between hepatocytes and incubation medium can be evaluated using the following published data [30]–[33]. It was shown using radioactive methionine distribution that methionine transport in hepatocytes is fast, passive, reversible and that the equilibrium between intracellular and extracellular methionine is established within a few minutes (Figures 3–5 in [33]). Moreover, these data demonstrate a linear fit between intracellular and extracellular methionine concentrations in the range from 18 µM to 10 mM (Figure 5 in [33]). In hepatocytes, methionine is transported via a Na^+^-dependent and a Na^+^-independent transporter, with the latter accounting for ∼90% of the transport at normal methionine concentrations, and the activity of transport is high enough to provide equilibration of extracellular and intracellular methionine within a few minutes (Table 2 in [32]), which confirms the data published in [33]. The kinetics of methionine transport in hepatocytes shows that at physiologically relevant concentration of methionine (100 µM) and at 37°C the equilibrium is achieved in 1 min (Figure 3 in [30]). The distribution of methionine between hepatocytes and the medium can be estimated using the data presented in Figure 3 of [30] as follows: at 100 µM [Met] in the medium, the equilibrium [Met] inside hepatocytes was 600 pmol/10^6^ cells. Based on the size of hepatocytes (see below in Metabolite Analysis), 10^6^ cells are estimated to have a total volume of 10 µl. This yields an intracellular [Met] of 60 µmol/l cells. Recalculating per volume of intracellular water, the intracellular [Met] is estimated to be ∼80 µM yielding a ratio between intra- and extra-cellular [Met] of 0.8. Similar calculations for the data presented in Figure 5 of [33] reveal that the amino acid remains equally distributed between the extracellular medium and inside cells at methionine concentrations ranging from 18 µM to 10 mM. It has also been shown *in vivo* that [Met] in blood plasma and in normal liver are fairly similar (Table 2 in [31]). Additionally, we note that preliminary experiments based on direct methionine measurements confirm fast and uniform distribution of methionine between hepatocytes and the incubation medium in concentration range from 40 to 400 µM. So we assume that extracellular and intracellular methionine concentrations are equal.

To incorporate the kinetics of folate metabolites into the model, specifically 5,10-methylenetetrahydrofolate (5,10-CH_2_-THF) and MTHF, which regulate MS, GNMT, and MTHFR activities, we take into account that most folate derivatives are interconnected via highly active and reversible enzymatic reactions [34],[35]. This allows us to consider a general folate pool with equilibrium ratios between its components for all folates except dihydrofolate (DHF) and MTHF. The last two metabolites are produced from 5,10-CH_2_-THF in irreversible reactions with low activity [36],[37]. Because of the equilibrium between components of folate pool we assume that concentration of 5,10-CH_2_-THF always constitutes 20% of the total pool as seen under normal physiological conditions [38],[39]. The total intracellular concentration of all folates is assumed to be constant, and DHF is not taken into account because we assume that its concentration is constant. Texts S1, S2, and S3 and Tables S1, S2, and S3 contain a detailed description of the model and its analysis.

### Hepatocyte Preparation

The experimental protocol was approved by the Scientific Council of the National Research Center for Hematology (NRCH). Female CBF1 mice (20–22 g) were obtained from the laboratory animal nursery in Stolbovaya, Moscow region, Russia. They were housed in a vivarium at the NRCH and provided with food and water *ad libitum*. Hepatocytes were isolated under sodium thiopental narcosis (i.p. injection of 5 mg/mouse). Liver was perfused for 5–10 min through the vena porta with a solution containing 115 mM NaCl, 5 mM KCl, 1 mM KH_2_PO_4_, 0.5 mM EGTA, 10 mM glucose, and 25 mM HEPES buffer, pH 7.5, saturated with 95% O_2_ and 5% CO_2_ at 37°C, followed by 7–8 min perfusion with the same solution lacking EGTA and containing 2.5 mM CaCl_2_ and 20–30 µg/ml of a collagenase-protease mixture (Liberase Blendzyme 3, Roche). The rate of perfusion was 7 ml/min. Then, the liver was dispersed at room temperature in the initial perfusing solution lacking EGTA. Hepatocytes were filtered through a 50 µm nylon mesh, sedimented by centrifugation and washed 3 times in 45 ml dispersing solution supplemented with 1 mM CaCl_2_, 1 mM MgCl_2_, 40 µM methionine and 2% BSA. Cell concentration and viability were determined using a hemocytometer after staining with 0.4% trypan blue. Cell preparations with viability ≥85% were used in all experiments. Hepatocytes were resuspended in the washing solution to a concentration of 1·10^6^ viable cells/ml, then 5 ml suspension aliquots were placed in 50 ml Ehrlenmeyer flasks and agitated at the rate of 100 RPM under an atmosphere of humidified 95% O_2_ and 5% CO_2_ at 37°C. At the beginning of the incubation, methionine was added to flasks to a final concentration of 40–400 µM. Cells prepared from one animal were incubated simultaneously in several flasks at different initial methionine concentrations for ∼3 h for a single experiment. Typically, the initial methionine concentrations were 40 µM, 400 µM, and two intermediate values.

### Metabolite Analysis

At the desired time intervals aliquots of cell suspension were collected from flasks and mixed with 0.2 volumes of 30% trichloroacetic acid. After centrifugation the supernatant was used for analysis of AdoMet, AdoHcy and methionine [18],[30].

The concentration of AdoMet and AdoHcy were measured by HPLC using an 8 µm Chromasil-100 C18 column, 250×4.6 mm, (Elsico, Russia) under isocratic conditions at a flow rate of 1 ml/min with monitoring at 254 nm. The mobile phase contained 40 mM NaH_2_PO_4_, 6 mM heptanesulfonic acid sodium salt (Sigma), and 15% methanol, pH 4. AdoHcy and AdoMet eluted as single peaks with retention times of 7 and 13 min respectively. The concentrations of AdoMet and AdoHcy in the samples were determined using calibration curves generated for each compound and normalized to the cell count in the suspension (i.e., expressed as µmoles/l cells). Based on an estimated cell diameter of 25–30 µm, the calculated value for the cell volume is 1·10^−11^ l.

To measure methionine concentration, the supernatant obtained after protein precipitation was derivatized with 2,4-dinitrofluorobenzene [30] and analyzed by HPLC using a 5 µm Diaspher-110 ODS column, 250×4 mm (BioChemMack, Russia) under isocratic conditions at a flow rate of 1 ml/min with monitoring at 355 nm. The mobile phase contained 45% acetonitrile in 1% acetic acid in water. The retention time of derivatized methionine was 12 min. The concentration of methionine in the samples was determined using a calibration curve and expressed as µmoles/l of suspension. The change in methionine concentration, due to intracellular metabolism in cell suspension over time was approximated by linear kinetics. The slope of the corresponding line and its deviation were determined using the Microcal Origin 6.0 software (Microcal Software, Inc.). To determine the rate of methionine consumption in hepatocytes, the slope was normalized to the total cell volume in suspension as described above.

As it was mentioned above, methionine transport in hepatocytes is passive, reversible and very fast over a wide range of [Met] (from 18 µM to 10 mM) and equilibrium between extracellular and intracellular methionine concentrations can be established within 1 min [30],[32],[33]. The equilibrium extracellular and intracellular methionine concentrations are similar in hepatocyte suspensions and in normal liver [30],[31],[33]. Thus, under our experimental conditions the total methionine concentration in cell suspension represents both medium and intracellular methionine concentration, and the rate of decrease in methionine concentration in suspension represents the rate of metabolic methionine consumption (catabolism) by the cells.

### Accession Numbers

The accession numbers of enzymes used in the model are defined in Text S1.

## Supporting Information

Figure S1 Kinetic scheme for reaction mechanism of MATIII containing two identical-subunits each with a catalytic and an allosteric site. The thick and thin arrows indicate reversible and irreversible steps, respectively. S, P, and M denote substrate, product, and effector and E, E-S, and E-2S denote free and different substrate-bound forms of enzyme, respectively. Parameter β determines cooperative effect of substrate binding to enzyme. Asterisks indicate enzyme with effector bound to one or two subunits. K_S1_, K_S2_, K_S3_ denote dissociation constants of enzyme-substrate complexes. k_f1_, k_f2_, k_f3_ denote rate constants of product formation. K_M_ denotes dissociation constant for effector in allosteric site. Coefficients 2 and 0.5 are statistical factors reflecting dimeric structure of the enzyme (see Hofmeyr JH, Cornish-Bowden A (1997) The reversible Hill equation: how to incorporate cooperative enzymes into metabolic models. Comput Appl Biosci 13: 377–385).
(0.87 MB TIF)

Figure S2Simulation of experimental MATIII kinetic data using the equation for the MATIIIreaction rate described in this study. Symbols show experimental data obtained in (Sullivan DM, Hoffman JL (1983) Fractionation and kinetic properties of rat liver and kidney methionine adenosyltransferase isozymes. Biochemistry 22: 1636–1641) *in vitro* with purified rat MATIII at [AdoMet] of 0 µM (square), 50 µM (triangle), 200 µM (circle), and 500 µM (inverted triangle). The corresponding curves shown by solid, dash, dot, and dash dot lines were calculated using Equation S18 and the following parameter values: 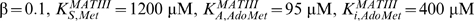.(0.14 MB TIF)

Figure S3 The predicted dependence of steady-state AdoMet production (dashed line) and consumption (solid line) rates on [AdoMet], at different [Met] of (A) 50 µM, (B) 80 µM, and (C) 100 µM. Points 1 and 3 correspond to stable steady states, while point 2 corresponds to unstable steady state. Curves were calculated at MATI activity of 1 mmol/h·l cells. All other parameter values are given in Tables S1 and S2. The insets show intersections of the curves in greater detail.
(0.26 MB TIF)

Figure S4 The dependence of (A) steady-state [AdoMet] and (B) transsulfuration/transmethylation ratio on [Met] in the model. Solid and dashed lines show points corresponding to stable and unstable steady states respectively. Circles (points 1 and 2) indicate steady states corresponding to lower and upper bounds of [Met], limiting the area of existence of multiple steady states. Arrows indicate movement of the steady-state point in case of increase in [Met] for point 1 or decrease in [Met] for point 2. Curves were calculated at MATI activity of 1 mmol/h·l cells. All other parameter values are given in Tables S1 and S2.
(0.25 MB TIF)

Figure S5 The dependence of steady-state [AdoMet] on [Met] in the model, calculated at different MATI activity. MATI activity in mmol/h·l cells is indicated above the corresponding curves. The solid line represents the curve shown in Figure 3A. All parameter values except MATI activity used for the calculations are given in Tables S1 and S2.
(0.19 MB TIF)

Table S1 Values of enzyme kinetic parameters in the model.
(0.29 MB DOC)

Table S2 Concentrations of metabolites assumed to be constant in the model.
(0.08 MB DOC)

Table S3 Normal physiological steady-state values of variables in the model.
(0.09 MB DOC)

Text S1 Mathematical model description.
(0.21 MB DOC)

Text S2 Description of the equation for the MATIII reaction rate.
(0.12 MB DOC)

Text S3 Mechanism of bistability in the model.
(0.03 MB DOC)
