# Supplementary figures and images for: An Allosteric Mechanism for Switching between Parallel Tracks in Mammalian Sulfur Metabolism
Source: PLoS Comput Biol. 2008 May 2;4(5):e1000076. doi: 10.1371/journal.pcbi.1000076 (PMC2346559; doi:10.1371/journal.pcbi.1000076)

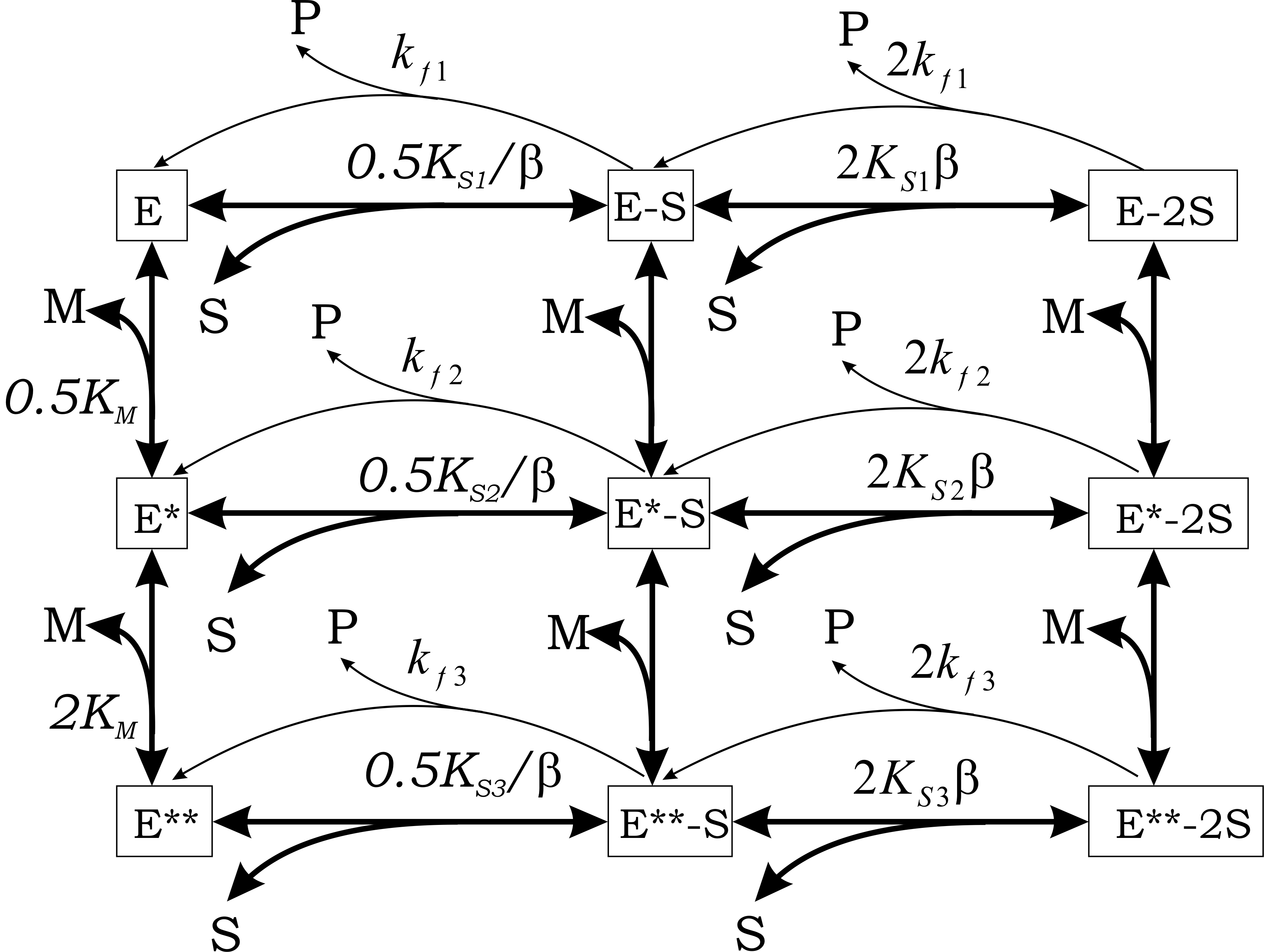

Supplement: Figure S1 — Kinetic scheme for reaction mechanism of MATIII containing two identical-subunits each with a catalytic and an allosteric site. The thick and thin arrows indicate reversible and irreversible steps, respectively. S, P, and M denote substrate, product, and effector and E, E-S, and E-2S denote free and different substrate-bound forms of enzyme, respectively. Parameter β determines cooperative effect of substrate binding to enzyme. Asterisks indicate enzyme with effector bound to one or two subunits. KS1, KS2, KS3 denote dissociation constants of enzyme-substrate complexes. kf1, kf2, kf3 denote rate constants of product formation. KM denotes dissociation constant for effector in allosteric site. Coefficients 2 and 0.5 are statistical factors reflecting dimeric structure of the enzyme (see Hofmeyr JH, Cornish-Bowden A (1997) The reversible Hill equation: how to incorporate cooperative enzymes into metabolic models. Comput Appl Biosci 13: 377–385). (0.87 MB TIF) [file pcbi.1000076.s001.tif]
