# Supplementary material for: An Allosteric Mechanism for Switching between Parallel Tracks in Mammalian Sulfur Metabolism
Source: PLoS Comput Biol. 2008 May 2;4(5):e1000076. doi: 10.1371/journal.pcbi.1000076 (PMC2346559; doi:10.1371/journal.pcbi.1000076)
Supplement: Table S1 — Values of enzyme kinetic parameters in the model. (0.29 MB DOC) [file pcbi.1000076.s006.doc]

Table S1

# Values of enzyme kinetic parameters in the model^a^.

| Parameter (units) | Model | Experiment | References and organism or tissue |
| --- | --- | --- | --- |
| MAT I | | | |
| $A^{MATI}$ (mmol/h/l cells) | 2.2 | - |  |
| $K_{m,Met}^{MATI}$ (μM) | 25 | 14 - 720 | [1–4] rat liver |
| $K_{i,AdoMet}^{MATI}$ (μM) | 25^b^ | 110 - 250 | [4,5] rat liver |
| **MAT III** | | | |
| $A_{0}^{MATIII}$ (mmol/h/l cells) | 15.8 | - |  |
| β | 0.1 | - | Calculated from [4]; rat liver |
| $K_{S,Met}^{MATIII}$ (μM) | 1200 | - |
| $K_{A,AdoMet}^{MATIII}$ (μM) | 50 | - |
| $K_{i,AdoMet}^{MATIII}$ (μM) | 700 | - |
| A^MATI^+A^MATIII^  (mmol/h/l cells) | 18 | 0.43 - 18 | [1,6] rat liver |
| **Methionine consumption in protein turnover** | | | |
| $A^{prot}$ (mmol/h/l cells) | 0.188 | 0.188 | Calculated from [7]; rat liver |
| $K_{m}^{prot}$ (μM) | 18 | 18 | [8] *T. thermophilus* |
| **Functional methylases** | | | |
| $A^{Meth}$ (mmol/h/l cells) | 13 | - |  |
| $K_{m,AdoMet}^{Meth}$ (μM),  at 35 μM AdoHcy | 9.8 | 7 – 30 | [9] rat  [10] rat |
| $K_{m,MGA}^{Meth}/[MGA]$ | 10 | 4.0 – 9.4 | [9] rat recombinant |
| $K_{i,AdoHcy}^{Meth}$ (μM) | 4.0 | 0.6 - 280 | [9] rat  [10] rat liver |
| **GNMT** | | | |
| $A^{GNMT}$ (mmol/h/l cells) | 60 | 0.7 – 60 | [11–17] rat liver |
| $K_{m,AdoMet}^{GNMT}$ (μM)  S_0.5,AdoMet_ (μM) | 350  586 | -  30 – 2000 | [13,18,19] rat liver |
| $K_{i,AdoHcy}^{GNMT}$ (μM)  I_0.5,AdoHcy_ (μM) | 52  18 | -  15 - 20 | [11] rabbit liver |
| $K_{i,MTHF}^{GNMT}$ (μM) | 0.3 | 0.3 | [19] rat liver |
| **AHC** | | | |
| *A^AHC^* (mmol/h/l cells) | - | 120 – 400 | [20,21] rat liver |
| *K^AHC^* (μM) | 1.4 | 1.4 | [21] rat liver |
| **MS** | | | |
| $A^{MS}$ (mmol/h/l cells) | 6.6^c^ | 1 – 3.3 | [22–24] rat liver |
| $K_{m,MTHF}^{MS}$ (μM) | 89 | 89 | [25] rat |
| $K_{m,Hcy}^{MS}$ (μM) | 1 | 1 – 3 | [26] *E.coli*  [27] pig liver  [25] rat liver |
| $K_{d,MTHF}^{MS}$ (μM) | 142 | 142 | [27] pig liver |
| BHMT | | | |
| $A^{BHMT}$ (mmol/h/l cells) | 60^c^ | 6 – 35 | [28–30] rat liver |
| $K_{m,Hcy}^{BHMT}$ (μM) | 12 | 12 – 32 | [31] rat liver  [32] pig liver |
| $K_{i,Met}^{BHMT}$ (μM) | 1.5 | - | Calculated from [31]; rat liver |
| $K_{i,DMG}^{BHMT}$ (μM) | 2 | - | Calculated from [31]; rat liver |
| CBS | | | |
| $A_{0}^{CBS}$ (mmol/h/l cells) | 120 | 10 - 188 | [33–35] rat liver |
| $K_{m,Serine}^{CBS}$ (mM) | 2.5 | 2 – 4 | [33,35] rat liver |
| $K_{m,Hcy}^{CBS}$ (mM) | 1 | 1 – 25 | [33,35] rat liver  [36] human liver |
| $K_{d,Serine}^{CBS}$ (μM) | 1 | - |  |
| $K_{A2}^{CBS}$ | 2 | 2 – 4 | [37,38] human |
| $K_{A1}^{CBS}$ (μM) | 60^d^ | 34 | [37] human |
| **MTHFR** | | | |
| $A_{0}^{MTHFR}$(mmol/h/l cells) | 4 | 1.3 – 4.2 | [23,39] rat liver |
| $K_{i0,AdoMet}^{MTHFR}$ (μM) | 3 | 0.9 – 3 | [40,41] pig liver |
| $K_{i,AdoHcy}^{MTHFR}$ (μM) | 3 | 3 | [42] pig liver |
| $K_{m,NADPH}^{MTHFR}$ (μM) | 16 | 16 – 28 | [22,43,44] pig liver |
| $K_{m,5,10-CH_{2}-THF}^{MTHFR}$ (μM) | 88 | 75 – 88 | [22,43–45] pig liver |
| $K_{i,MTHF}^{MTHFR}$ (μM) | 20 | 20 | [45] pig liver |

^a^ Most parameter values in the Table S1 were obtained for rodent enzymes. If data for rodent enzymes were not available, we used parameter values obtained for other species.

^b^ The value of $K_{i,AdoMet}^{MATI}$ was adjusted in order to obtain the best fit of experimental dependence of [AdoMet] on [Met].

^c^ Activities of MS and BHMT were increased in the model compared to literature data to get transmethylation/transsulfuration flux ratio (V_MS_ + V_BHMT_) / V_CBS_ close to experimentally measured value of 1.0 [46] at normal physiological metabolite concentrations.

^d^ The value of $K_{A1}^{CBS}$was adjusted in order to obtain the best fit of experimental dependence of [AdoHcy] on [Met].

**REFERENCES**

1. Kunz GL, Hoffman JL, Chia CS, Stremel B (1980) Separation of rat liver methionine adenosyltransferase isozymes by hydrophobic chromatography. Arch Biochem Biophys 202: 565-572.

2. Okada G, Teraoka H, Tsukada K (1981) Multiple species of mammalian S-adenosylmethionine synthetase. Partial purification and characterization. Biochemistry 20: 934-940.

3. Pajares MA, Duran C, Corrales F, Mato JM (1994) Protein kinase C phosphorylation of rat liver S-adenosylmethionine synthetase: dissociation and production of an active monomer. Biochem J 303 ( Pt 3): 949-955.

4. Sullivan DM, Hoffman JL (1983) Fractionation and kinetic properties of rat liver and kidney methionine adenosyltransferase isozymes. Biochemistry 22: 1636-1641.

5. Cabrero C, Puerta J, Alemany S (1987) Purification and comparison of two forms of S-adenosyl-L-methionine synthetase from rat liver. Eur J Biochem 170: 299-304.

6. Lu SC, Huang ZZ, Yang H, Mato JM, Avila MA, Tsukamoto H (2000) Changes in methionine adenosyltransferase and S-adenosylmethionine homeostasis in alcoholic rat liver. Am J Physiol Gastrointest Liver Physiol 279: G178-G185.

7. Elsner P, Dich J, Grunnet N (1994) Quantification of protein turnover in primary cultures of rat hepatocytes. Biochim Biophys Acta 1199: 157-165.

8. Nureki O, Kohno T, Sakamoto K, Miyazawa T, Yokoyama S (1993) Chemical modification and mutagenesis studies on zinc binding of aminoacyl-tRNA synthetases. J Biol Chem 268: 15368-15373.

9. Takata Y, Konishi K, Gomi T, Fujioka M (1994) Rat guanidinoacetate methyltransferase. Effect of site-directed alteration of an aspartic acid residue that is conserved across most mammalian S-adenosylmethionine-dependent methyltransferases. J Biol Chem 269: 5537-5542.

10. Ridgway ND, Vance DE (1988) Kinetic mechanism of phosphatidylethanolamine N-methyltransferase. J Biol Chem 263: 16864-16871.

11. Kerr SJ (1972) Competing methyltransferase systems. J Biol Chem 247: 4248-4252.

12. Nieman KM, Rowling MJ, Garrow TA, Schalinske KL (2004) Modulation of methyl group metabolism by streptozotocin-induced diabetes and all-trans-retinoic acid. J Biol Chem 279: 45708-45712.

13. Ogawa H, Fujioka M (1982) Purification and properties of glycine N-methyltransferase from rat liver. J Biol Chem 257: 3447-3452.

14. Rowling MJ, Schalinske KL (2001) Retinoid compounds activate and induce hepatic glycine N-methyltransferase in rats. J Nutr 131: 1914-1917.

15. Rowling MJ, McMullen MH, Chipman DC, Schalinske KL (2002) Hepatic glycine N-methyltransferase is up-regulated by excess dietary methionine in rats. J Nutr 132: 2545-2550.

16. Rowling MJ, McMullen MH, Schalinske KL (2002) Vitamin A and its derivatives induce hepatic glycine N-methyltransferase and hypomethylation of DNA in rats. J Nutr 132: 365-369.

17. Rowling MJ, Schalinske KL (2003) Retinoic acid and glucocorticoid treatment induce hepatic glycine N-methyltransferase and lower plasma homocysteine concentrations in rats and rat hepatoma cells. J Nutr 133: 3392-3398.

18. Ogawa H, Gomi T, Takusagawa F, Fujioka M (1998) Structure, function and physiological role of glycine N-methyltransferase. Int J Biochem Cell Biol 30: 13-26.

19. Yeo EJ, Briggs WT, Wagner C (1999) Inhibition of glycine N-methyltransferase by 5-methyltetrahydrofolate pentaglutamate. J Biol Chem 274: 37559-37564.

20. Kajander EO, Raina AM (1981) Affinity-chromatographic purification of S-adenosyl-L-homocysteine hydrolase. Some properties of the enzyme from rat liver. Biochem J 193: 503-512.

21. de la Haba G, Cantoni GL (1959) The enzymatic synthesis of S-adenosyl-L-homocysteine from adenosine and homocysteine. J Biol Chem 234: 603-608.

22. Huang L, Zhang J, Hayakawa T, Tsuge H (2001) Assays of methylenetetrahydrofolate reductase and methionine synthase activities by monitoring 5-methyltetrahydrofolate and tetrahydrofolate using high-performance liquid chromatography with fluorescence detection. Anal Biochem 299: 253-259.

23. Stead LM, Au KP, Jacobs RL, Brosnan ME, Brosnan JT (2001) Methylation demand and homocysteine metabolism: effects of dietary provision of creatine and guanidinoacetate. Am J Physiol Endocrinol Metab 281: E1095-E1100.

24. Yamada K, Kawata T, Wada M, Isshiki T, Onoda J, Kawanishi T, Kunou A, Tadokoro T, Tobimatsu T, Maekawa A, Toraya T (2000) Extremely low activity of methionine synthase in vitamin B-12-deficient rats may be related to effects on coenzyme stabilization rather than to changes in coenzyme induction. J Nutr 130: 1894-1900.

25. Yamada K, Yamada S, Tobimatsu T, Toraya T (1999) Heterologous high level expression, purification, and enzymological properties of recombinant rat cobalamin-dependent methionine synthase. J Biol Chem 274: 35571-35576.

26. Banerjee RV, Frasca V, Ballou DP, Matthews RG (1990) Participation of cob(I) alamin in the reaction catalyzed by methionine synthase from Escherichia coli: a steady-state and rapid reaction kinetic analysis. Biochemistry 29: 11101-11109.

27. Chen Z, Crippen K, Gulati S, Banerjee R (1994) Purification and kinetic mechanism of a mammalian methionine synthase from pig liver. J Biol Chem 269: 27193-27197.

28. Davis CD, Uthus EO (2003) Dietary folate and selenium affect dimethylhydrazine-induced aberrant crypt formation, global DNA methylation and one-carbon metabolism in rats. J Nutr 133: 2907-2914.

29. Forestier M, Banninger R, Reichen J, Solioz M (2003) Betaine homocysteine methyltransferase: gene cloning and expression analysis in rat liver cirrhosis. Biochim Biophys Acta 1638: 29-34.

30. Uthus EO, Yokoi K, Davis CD (2002) Selenium deficiency in Fisher-344 rats decreases plasma and tissue homocysteine concentrations and alters plasma homocysteine and cysteine redox status. J Nutr 132: 1122-1128.

31. Finkelstein JD, Harris BJ, Kyle WE (1972) Methionine metabolism in mammals: kinetic study of betaine-homocysteine methyltransferase. Arch Biochem Biophys 153: 320-324.

32. Garrow TA (1996) Purification, kinetic properties, and cDNA cloning of mammalian betaine-homocysteine methyltransferase. J Biol Chem 271: 22831-22838.

33. Borcsok E, Abeles RH (1982) Mechanism of action of cystathionine synthase. Arch Biochem Biophys 213: 695-707.

34. Ratnam S, Maclean KN, Jacobs RL, Brosnan ME, Kraus JP, Brosnan JT (2002) Hormonal regulation of cystathionine beta-synthase expression in liver. J Biol Chem 277: 42912-42918.

35. Skovby F, Kraus JP, Rosenberg LE (1984) Biosynthesis and proteolytic activation of cystathionine beta-synthase in rat liver. J Biol Chem 259: 588-593.

36. Kraus JP, Rosenberg LE (1983) Cystathionine beta-synthase from human liver: improved purification scheme and additional characterization of the enzyme in crude and pure form. Arch Biochem Biophys 222: 44-52.

37. Bukovska G, Kery V, Kraus JP (1994) Expression of human cystathionine beta-synthase in Escherichia coli: purification and characterization. Protein Expr Purif 5: 442-448.

38. Taoka S, Ohja S, Shan X, Kruger WD, Banerjee R (1998) Evidence for heme-mediated redox regulation of human cystathionine beta-synthase activity. J Biol Chem 273: 25179-25184.

39. Jacobs RL, Stead LM, Brosnan ME, Brosnan JT (2001) Hyperglucagonemia in rats results in decreased plasma homocysteine and increased flux through the transsulfuration pathway in liver. J Biol Chem 276: 43740-43747.

40. Sumner J, Jencks DA, Khani S, Matthews RG (1986) Photoaffinity labeling of methylenetetrahydrofolate reductase with 8-azido-S-adenosylmethionine. J Biol Chem 261: 7697-7700.

41. Jencks DA, Mathews RG (1987) Allosteric inhibition of methylenetetrahydrofolate reductase by adenosylmethionine. Effects of adenosylmethionine and NADPH on the equilibrium between active and inactive forms of the enzyme and on the kinetics of approach to equilibrium. J Biol Chem 262: 2485-2493.

42. Matthews RG, Daubner SC (1982) Modulation of methylenetetrahydrofolate reductase activity by S-adenosylmethionine and by dihydrofolate and its polyglutamate analogues. Adv Enzyme Regul 20: 123-131.

43. Daubner SC, Matthews RG (1982) Purification and properties of methylenetetrahydrofolate reductase from pig liver. J Biol Chem 257: 140-145.

44. Vanoni MA, Ballou DP, Matthews RG (1983) Methylenetetrahydrofolate reductase. Steady state and rapid reaction studies on the NADPH-methylenetetrahydrofolate, NADPH-menadione, and methyltetrahydrofolate-menadione oxidoreductase activities of the enzyme. J Biol Chem 258: 11510-11514.

45. Vanoni MA, Matthews RG (1984) Kinetic isotope effects on the oxidation of reduced nicotinamide adenine dinucleotide phosphate by the flavoprotein methylenetetrahydrofolate reductase. Biochemistry 23: 5272-5279.

46. Finkelstein JD, Martin JJ (1984) Methionine metabolism in mammals. Distribution of homocysteine between competing pathways. J Biol Chem 259: 9508-9513.
