# Supplementary material for: An Allosteric Mechanism for Switching between Parallel Tracks in Mammalian Sulfur Metabolism
Source: PLoS Comput Biol. 2008 May 2;4(5):e1000076. doi: 10.1371/journal.pcbi.1000076 (PMC2346559; doi:10.1371/journal.pcbi.1000076)
Supplement: Table S2 — Concentrations of metabolites assumed to be constant in the model. (0.08 MB DOC) [file pcbi.1000076.s007.doc]

Table S2

Concentrations of metabolites assumed to be constant in the model.

Experimental values summarize data obtained in rat liver

| Metabolite (μM) | Model | Experiment | References |
| --- | --- | --- | --- |
| Adenosine | 14 | 0.4-50 | [1,2] |
| ATP | 2000 | 2000 | [3] |
| Betaine | 1900 | 220 - 7300 | [4–6] |
| Dimethylglycine | 100 | 10 - 250 | [5] |
| F_0_ (total folates concentration) | 25 | 18 - 28 | [7,8] |
| Glycine | 1400 | 1200-1800 | [9,10] |
| NADPH | 200 | 200 - 300 | [11,12] |
| Serine | 590 | 140 - 1800 | [4] |

# REFERENCES

1. Bontemps F, Vincent MF, Van den BG (1993) Mechanisms of elevation of adenosine levels in anoxic hepatocytes. Biochem J 290 ( Pt 3): 671-677.

2. Chagoya dS, V, Hernandez-Munoz R, Sanchez L, Vidrio S, Yanez L, Suarez J (1991) Twenty-four-hour changes of S-adenosylmethionine, S-adenosylhomocysteine adenosine and their metabolizing enzymes in rat liver; possible physiological significance in phospholipid methylation. Int J Biochem 23: 1439-1443.

3. Chen L, Zeng Y, Yang H, Lee TD, French SW, Corrales FJ, Garcia-Trevijano ER, Avila MA, Mato JM, Lu SC (2004) Impaired liver regeneration in mice lacking methionine adenosyltransferase 1A. FASEB J 18: 914-916.

4. Finkelstein JD, Martin JJ (1986) Methionine metabolism in mammals. Adaptation to methionine excess. J Biol Chem 261: 1582-1587.

5. Garrow TA (1996) Purification, kinetic properties, and cDNA cloning of mammalian betaine-homocysteine methyltransferase. J Biol Chem 271: 22831-22838.

6. Millian NS, Garrow TA (1998) Human betaine-homocysteine methyltransferase is a zinc metalloenzyme. Arch Biochem Biophys 356: 93-98.

7. Chanson A, Sayd T, Rock E, Chambon C, Sante-Lhoutellier V, Potier de CG, Brachet P (2005) Proteomic analysis reveals changes in the liver protein pattern of rats exposed to dietary folate deficiency. J Nutr 135: 2524-2529.

8. Horne DW (2003) Neither methionine nor nitrous oxide inactivation of methionine synthase affect the concentration of 5,10-methylenetetrahydrofolate in rat liver. J Nutr 133: 476-478.

9. Scheer JB, Mackey AD, Gregory JF, III (2005) Activities of hepatic cytosolic and mitochondrial forms of serine hydroxymethyltransferase and hepatic glycine concentration are affected by vitamin B-6 intake in rats. J Nutr 135: 233-238.

10. Regina M, Korhonen VP, Smith TK, Alakuijala L, Eloranta TO (1993) Methionine toxicity in the rat in relation to hepatic accumulation of S-adenosylmethionine: prevention by dietary stimulation of the hepatic transsulfuration pathway. Arch Biochem Biophys 300: 598-607.

11. Jencks DA, Mathews RG (1987) Allosteric inhibition of methylenetetrahydrofolate reductase by adenosylmethionine. Effects of adenosylmethionine and NADPH on the equilibrium between active and inactive forms of the enzyme and on the kinetics of approach to equilibrium. J Biol Chem 262: 2485-2493.

12. Vanoni MA, Matthews RG (1984) Kinetic isotope effects on the oxidation of reduced nicotinamide adenine dinucleotide phosphate by the flavoprotein methylenetetrahydrofolate reductase. Biochemistry 23: 5272-5279.
