# Supplementary material for: An Allosteric Mechanism for Switching between Parallel Tracks in Mammalian Sulfur Metabolism
Source: PLoS Comput Biol. 2008 May 2;4(5):e1000076. doi: 10.1371/journal.pcbi.1000076 (PMC2346559; doi:10.1371/journal.pcbi.1000076)
Supplement: Table S3 — Normal physiological steady-state values of variables in the model. (0.09 MB DOC) [file pcbi.1000076.s008.doc]

# Table S3

# Normal physiological steady-state values of variables in the model.

| Metabolite (μM) | Model | Experiment | References |
| --- | --- | --- | --- |
| Met | 50 | 20-75 | [1–3] rat liver |
| AdoMet | 60 | 50-170 | [1,2] rat liver |
| AdoHcy | 35 | 3-40 | [1,2] rat liver |
| Hcy | 3.5 | 3-6 | [4] mouse liver |
| MTHF | 1.7 | 1-16 | [5–8] rat liver |
| 5,10-CH_2_-THF | 4.7 | 4-8 | [6] rat liver |
| F^a^ | 23.3 | 23.3 | Calculated from [5–8] rat liver |

^a^*F* denotes the pool of folates interconnected via highly active reversible enzymatic reactions (see description to Eq. S1)

## REFERENCES

1. Finkelstein JD, Kyle WE, Harris BJ, Martin JJ (1982) Methionine metabolism in mammals: concentration of metabolites in rat tissues. J Nutr 112: 1011-1018.

2. Finkelstein JD, Martin JJ (1986) Methionine metabolism in mammals. Adaptation to methionine excess. J Biol Chem 261: 1582-1587.

3. Jacobs RL, Stead LM, Brosnan ME, Brosnan JT (2001) Hyperglucagonemia in rats results in decreased plasma homocysteine and increased flux through the transsulfuration pathway in liver. J Biol Chem 276: 43740-43747.

4. Vitvitsky V, Prudova A, Stabler S, Dayal S, Lentz SR, Banerjee R (2007) Testosterone Regulation of Renal Cystathionine {beta}-synthase. Implications for Sex-dependent Differences in Plasma Homocysteine Levels. Am J Physiol Renal Physiol

5. Chanson A, Sayd T, Rock E, Chambon C, Sante-Lhoutellier V, Potier de CG, Brachet P (2005) Proteomic analysis reveals changes in the liver protein pattern of rats exposed to dietary folate deficiency. J Nutr 135: 2524-2529.

6. Horne DW (2003) Neither methionine nor nitrous oxide inactivation of methionine synthase affect the concentration of 5,10-methylenetetrahydrofolate in rat liver. J Nutr 133: 476-478.

7. Ozias MK, Schalinske KL (2003) All-trans-retinoic acid rapidly induces glycine N-methyltransferase in a dose-dependent manner and reduces circulating methionine and homocysteine levels in rats. J Nutr 133: 4090-4094.

8. Taes YE, Delanghe JR, De Vriese AS, Rombaut R, Van CJ, Lameire NH (2003) Creatine supplementation decreases homocysteine in an animal model of uremia. Kidney Int 64: 1331-1337.
