# Supplementary material for: An Allosteric Mechanism for Switching between Parallel Tracks in Mammalian Sulfur Metabolism
Source: PLoS Comput Biol. 2008 May 2;4(5):e1000076. doi: 10.1371/journal.pcbi.1000076 (PMC2346559; doi:10.1371/journal.pcbi.1000076)
Supplement: Text S1 — Mathematical model description. (0.21 MB DOC) [file pcbi.1000076.s009.doc]

**Text S1. Mathematical model description**.

Model is represented by the following set of equations:

$$\frac{d[Met]}{dt}=\left(V^{influx}+(V^{BHMT}+V^{MS}-V^{MATI}-V^{MATIII}-V^{prot})w_{hep}\right)/\left(w_{hep}+w_{med}\right)$$

$\frac{d[AdoMet]}{dt}=V^{MATI}+V^{MATIII}-V^{Meth}-V^{GNMT}$ (Eq.S1)

$$\frac{d[AdoHcy]}{dt}=\left(V^{Meth}+V^{GNMT}-V^{CBS}-V^{BHMT}-V^{MS}\right)/\left(1+K^{AHC}/[Ado]\right)$$

$$[Hcy]=[AdoHcy]\frac{K^{AHC}}{[Ado]}$$

$$\frac{d[MTHF]}{dt}=V^{MTHFR}-V^{MS}$$

$$[MTHF]+[F]=F_{0}$$

$[5,10-CH_{2}-THF]=0.2[F]$

$$[Met]_{ext}=[Met]$$

Here *K^AHC^* is the equilibrium constant for the adenosylhomocysteinase reaction, Ado denotes adenosine, *V^MS^* and *V^MTHFR^* denote rates of the MS and MTHFR reactions, *F* denotes the pool of folates interconnected via highly active reversible enzymatic reactions, and *F_0_* denotes total concentration of all intracellular folates except DHF. Model variables are extracellular concentration of methionine and intracellular concentrations of methionine, AdoMet, AdoHcy, Hcy, 5,10-CH_2_-THF, MTHF and the folate pool (*F*).

In order to further simplify the model, we made additional general assumptions. Concentrations of ATP, adenosine, betaine, dimethylglycine, glycine, NADPH, and serine, as well as a total concentration of all intracellular folates (*F_0_*) are assumed to be constant. In this way, either there is no dependence of reaction rates on these metabolites, or they are included in the relevant equations as parameters. Concentrations of metabolites, which are constant in the model, are presented in Table S2.

Equations for the rates of particular enzymatic reactions are shown below and values of kinetic parameters of enzymes are presented in Table S1.

*MAT I (EC 2.5.1.6).* The equation for MATI reaction rate was taken from [1]. It includes the dependence of reaction rate on methionine as a substrate and product inhibition by AdoMet:

$V^{MATI}=\frac{A^{MATI}}{1+\frac{K_{m,Met}^{MATI}}{[Met]}\left(1+[AdoMet]/K_{i,AdoMet}^{MATI}\right)}$ (Eq.S2)

# *MAT III (EC 2.5.1.6).* The equation for MATIII reaction rate includes positive cooperative dependence on methionine [2] as well as activation and inhibition at different AdoMet concentration [2,3]. The development of the equation is described in the Text S2.

(Eq.S3)

*Methionine consumption in protein turnover.* We tried to estimate the amount of methionine consumed in hepatocytes in protein turnover. The rates of protein synthesis and degradation in hepatocytes reported in [4] are equal to 13.1 and 9.3 mg/h per g of protein, respectively. Taking into account that the mean molecular mass of amino acids in proteins is 110 [5], the frequency of methionine in eukaryotic proteins is 2% [6], and the protein content in rat liver is 200 g/l [7], we obtained rates for methionine incorporation into and release from proteins in liver, of 476 and 338 μmol/h•l tissue respectively. Thus, under normal conditions in hepatocytes, the net rate of methionine consumption in protein synthesis is 138 μmol/h•l cells. The first step in methionine consumption in protein synthesis is charging of Met-tRNA catalyzed by methionyl-tRNA-synthetase. We assumed that the dependence of methionine consumption for protein synthesis on methionine is determined by the dependence of methionyl-tRNA-synthetase on methionine [8].

$V^{prot}=A^{prot}\frac{[Met]}{[Met]+K_{m}^{prot}}$ (Eq.S4)

*Functional methylases.*We represented all intracellular methylases (except GNMT) catalyzing methyl group transfer from AdoMet to different methyl group acceptors as a single activity catalyzed by a generalized enzyme that transfers methyl groups to a generalized methyl group acceptor, MGA. The concentration of MGA, [MGA], is assumed to be a constant. The ratio of the corresponding Michaelis–Menten constant to [MGA] is used as a parameter. The equation for the rate of functional methylation reactions includes the dependence on both substrates (AdoMet and MGA) and inhibition by AdoHcy and was taken from [1].

$$V^{Meth}=\frac{A^{Meth}}{1+\frac{K_{m,AdoMet}^{Meth}}{[AdoMet]}+\frac{K_{m,MGA}^{Meth}}{[MGA]}\left(1+\frac{K_{m,AdoMet}^{Meth}}{[AdoMet]}\right)}$$

$K_{m,AdoMet}^{Meth}=1+[AdoHcy]/K_{i,AdoHcy}^{Meth}$ (Eq.S5)

G*NMT (EC 2.1.1.20).*The equation for the GNMT reaction rate includes a cooperative dependence on AdoMet with a Hill coefficient of 2.3 [9] as well as competitive inhibition by AdoHcy [10,11] and noncompetitive inhibition by MTHF [12].

$V^{GNMT}=\frac{A^{GNMT}}{1+\left[\frac{K_{m,AdoMet}^{GNMT}}{[AdoMet]}\left(1+\frac{[AdoHcy]}{K_{i,AdoHcy}^{GNMT}}\right)\right]^{2.3}} \cdot\frac{1}{1+\frac{[MTHF]}{K_{i,MTHF}^{GNMT}}}$ (Eq.S6)

*AHC (EC 3.3.1.1)*. This enzyme catalyses reversible reaction of AdoHcy hydrolysis with formation of Hcy and adenosine. Since AHC activity in hepatocytes greatly exceeds activities of other enzymes of methionine metabolism (Supporting Table 1), we assumed that AHC reaction is in equilibrium and used corresponding equation for homocysteine concentration (Eq.S1).

# *MS (EC 2.1.1.13).*This enzyme catalyses an irreversible reaction via an ordered bi-bi mechanism with MTHF as the first substrate and methionine as the first product [13,14]. The reaction rate can be described by equation S7 [15].

$V^{MS}=\frac{A^{MS}}{1+\frac{K_{m,MTHF}^{MS}}{[MTHF]}+\frac{K_{m,Hcy}^{MS}}{[Hcy]}\left(1+\frac{K_{d,MTHF}^{MS}}{[MTHF]}\right)}$ (Eq.S7)

# *BHMT (EC 2.1.1.5).* This enzyme catalyses an irreversible reaction via an ordered bi-bi mechanism with Hcy as the first substrate and dimethylglycine as the first product [16]. The enzyme is inhibited by both products, methionine and dimethyglycine [16] and the reaction rate can be described by equation S8 [15].

$V^{BHMT}=\frac{A^{BHMT}}{1+\frac{[DMG]}{K_{i,DMG}^{BHMT}}+\frac{K_{m,Hcy}^{BHMT}}{[Hcy]}\left(1+\frac{[Met]}{K_{i,Met}^{BHMT}}\right)}$ (Eq.S8)

# *CBS (EC 4.2.1.22).* The rate equation for the CBS reaction was taken from [17]. It includes the dependence of the reaction rate on substrates and activation by AdoMet:

$V^{CBS}=\frac{A^{CBS}}{1+\frac{K_{m,Serine}^{CBS}}{[Serine]}+\frac{K_{m,Hcy}^{CBS}}{[Hcy]}\left(1+\frac{K_{d,Serine}^{CBS}}{[Serine]}\right)}$ (Eq.S9)

$$A^{CBS}=A_{0}^{CBS}\left(1+\frac{K_{A2}^{CBS} -1}{1+K_{A1}^{CBS} /[AdoMet]}\right)$$

*MTHFR (EC 1.5.1.20).* This enzyme catalyses a ping-pong reaction with NADPH as the first substrate and MTHF as the second product [18–21]. The first step in the reaction mechanism (i.e. NADPH oxidation) is irreversible, while the second step (i.e. MTHF reduction) is reversible [21]. MTHFR is allosterically inhibited by AdoMet and exhibits 4.6% of residual enzyme activity under conditions of maximal inhibition [22]. This inhibition is diminished by AdoHcy [23,24]. To simulate this regulation in the model, we assumed that MTHFR has an allosteric site that can either bind AdoMet or AdoHcy. The binding of AdoMet diminishes enzyme activity whereas binding of AdoHcy does not change the kinetic parameters of the enzyme. We then included this regulation in the rate equation for a ping-pong mechanism [15]:

$V^{MTHFR}=\frac{A^{MTHFR}}{1+\frac{K_{m,NADPH}^{MTHFR}}{[NADPH]}\left(1+\frac{[MTHF]}{K_{i,MTHF}^{MTHFR}}\right)+\frac{K_{m,5,10-CH_{2}-THF}^{MTHFR}}{[5,10-CH_{2}-THF]}}$ (Eq.S10)

$$\begin{matrix} A^{MTHFR}=A_{0}^{MTHFR}\left(\frac{1}{1+[AdoMet]/K_{i,AdoMet}^{MTHFR}}+\frac{0.046}{1+K_{i,AdoMet}^{MTHFR}/[AdoMet]}\right) \\ K_{i,AdoMet}^{MTHFR}=K_{i0,AdoMet}^{MTHFR}\left(1+[AdoHcy]/K_{i,AdoHcy}^{MTHFR}\right) \end{matrix}$$

For numeric analysis of the mathematical model we used Matlab software with Matlab continuation toolbox [25] and DBSolve software [26]. We used the complete equation set (Eq.S1) for analysis of the steady-state dependence of methionine metabolism on methionine influx rate and for simulation of *in vitro* kinetics of change in AdoMet and Met concentrations in hepatocytes. Simulation of the *in vitro* kinetics of methionine metabolism was examined at *w_med_/w_hep_*=99 (i.e. 1% cell suspension) and V^influx^ = 0. All model variables except [Met] were set to physiological steady-state values (Table S3) and calculations were started at different initial [Met] values.

When calculating the steady-state dependence of methionine metabolism on methionine concentration, we excluded the first equation from the system (Eq.S1), and in the remaining equations [Met] was included as a parameter.

Analysis of the model shows that at a constant methionine concentration equal to the normal physiological value of 50 μM and at normal values of enzyme kinetic parameters (Table S1) and model parameters (Table S2) there is only one stable steady state with values for variables that are close to normal experimental concentrations for the corresponding metabolites (Table S3). Values of enzyme kinetic parameters listed in Table S1 provide the best description of results obtained in our experiments with suspension of murine hepatocytes.

**REFERENCES**

1. Martinov MV, Vitvitsky VM, Mosharov EV, Banerjee R, Ataullakhanov FI (2000) A substrate switch: a new mode of regulation in the methionine metabolic pathway. J Theor Biol 204: 521-532.

2. Sullivan DM, Hoffman JL (1983) Fractionation and kinetic properties of rat liver and kidney methionine adenosyltransferase isozymes. Biochemistry 22: 1636-1641.

3. Cabrero C, Puerta J, Alemany S (1987) Purification and comparison of two forms of S-adenosyl-L-methionine synthetase from rat liver. Eur J Biochem 170: 299-304.

4. Elsner P, Dich J, Grunnet N (1994) Quantification of protein turnover in primary cultures of rat hepatocytes. Biochim Biophys Acta 1199: 157-165.

5. Senko M, Beu S, McLafferty F (1995) Determination of Monoisotopic Masses and Ion Populations for Large Biomolecules from Resolved Isotopic Distributions. Journal of the American Society for Mass Spectrometry 6: 229-233.

6. Wu CH, Apweiler R, Bairoch A, Natale DA, Barker WC, Boeckmann B, Ferro S, Gasteiger E, Huang H, Lopez R, Magrane M, Martin MJ, Mazumder R, O'Donovan C, Redaschi N, Suzek B (2006) The Universal Protein Resource (UniProt): an expanding universe of protein information. Nucleic Acids Res 34: D187-D191.

7. Bella DL, Hahn C, Stipanuk MH (1999) Effects of nonsulfur and sulfur amino acids on the regulation of hepatic enzymes of cysteine metabolism. Am J Physiol 277: E144-E153.

8. Xu B, Krudy GA, Rosevear PR (1993) Identification of the metal ligands and characterization of a putative zinc finger in methionyl-tRNA synthetase. J Biol Chem 268: 16259-16264.

9. Ogawa H, Gomi T, Fujioka M (1993) Mammalian glycine N-methyltransferases. Comparative kinetic and structural properties of the enzymes from human, rat, rabbit and pig livers. Comp Biochem Physiol B 106: 601-611.

10. Heady JE, Kerr SJ (1973) Purification and characterization of glycine N-methyltransferase. J Biol Chem 248: 69-72.

11. Ogawa H, Fujioka M (1982) Purification and properties of glycine N-methyltransferase from rat liver. J Biol Chem 257: 3447-3452.

12. Yeo EJ, Briggs WT, Wagner C (1999) Inhibition of glycine N-methyltransferase by 5-methyltetrahydrofolate pentaglutamate. J Biol Chem 274: 37559-37564.

13. Banerjee RV, Frasca V, Ballou DP, Matthews RG (1990) Participation of cob(I) alamin in the reaction catalyzed by methionine synthase from Escherichia coli: a steady-state and rapid reaction kinetic analysis. Biochemistry 29: 11101-11109.

14. Chen Z, Crippen K, Gulati S, Banerjee R (1994) Purification and kinetic mechanism of a mammalian methionine synthase from pig liver. J Biol Chem 269: 27193-27197.

15. Cornish-Bowden A (1994) Fundamentals of Enzyme Kinetics. Portland Press Ltd.

16. Finkelstein JD, Harris BJ, Kyle WE (1972) Methionine metabolism in mammals: kinetic study of betaine-homocysteine methyltransferase. Arch Biochem Biophys 153: 320-324.

17. Prudova A, Martinov MV, Vitvitsky VM, Ataullakhanov FI, Banerjee R (2005) Analysis of pathological defects in methionine metabolism using a simple mathematical model. Biochim Biophys Acta 1741: 331-338.

18. Sheppard CA, Trimmer EE, Matthews RG (1999) Purification and properties of NADH-dependent 5, 10-methylenetetrahydrofolate reductase (MetF) from Escherichia coli. J Bacteriol 181: 718-725.

19. Trimmer EE, Ballou DP, Matthews RG (2001) Methylenetetrahydrofolate reductase from Escherichia coli: elucidation of the kinetic mechanism by steady-state and rapid-reaction studies. Biochemistry 40: 6205-6215.

20. Trimmer EE, Ballou DP, Ludwig ML, Matthews RG (2001) Folate activation and catalysis in methylenetetrahydrofolate reductase from Escherichia coli: roles for aspartate 120 and glutamate 28. Biochemistry 40: 6216-6226.

21. Vanoni MA, Matthews RG (1984) Kinetic isotope effects on the oxidation of reduced nicotinamide adenine dinucleotide phosphate by the flavoprotein methylenetetrahydrofolate reductase. Biochemistry 23: 5272-5279.

22. Sumner J, Jencks DA, Khani S, Matthews RG (1986) Photoaffinity labeling of methylenetetrahydrofolate reductase with 8-azido-S-adenosylmethionine. J Biol Chem 261: 7697-7700.

23. Jencks DA, Mathews RG (1987) Allosteric inhibition of methylenetetrahydrofolate reductase by adenosylmethionine. Effects of adenosylmethionine and NADPH on the equilibrium between active and inactive forms of the enzyme and on the kinetics of approach to equilibrium. J Biol Chem 262: 2485-2493.

24. Matthews RG, Daubner SC (1982) Modulation of methylenetetrahydrofolate reductase activity by S-adenosylmethionine and by dihydrofolate and its polyglutamate analogues. Adv Enzyme Regul 20: 123-131.

25. Dhooge A, Kuznetsov YuA (2003) MATCONT : A Matlab package for numerical bifurcation analysis of ODEs. ACM Transactions on Mathematical Software 29: 141-164.

26. Goryanin I, Hodgman TC, Selkov E (1999) Mathematical simulation and analysis of cellular metabolism and regulation. Bioinformatics 15: 749-758.
