# Supplementary material for: An Allosteric Mechanism for Switching between Parallel Tracks in Mammalian Sulfur Metabolism
Source: PLoS Comput Biol. 2008 May 2;4(5):e1000076. doi: 10.1371/journal.pcbi.1000076 (PMC2346559; doi:10.1371/journal.pcbi.1000076)
Supplement: Text S2 — Description of the equation for the MATIII reaction rate. (0.12 MB DOC) [file pcbi.1000076.s010.doc]

# Text S2. Description of the equation for the MATIII reaction rate.

MATIII consists of two identical subunits and exhibits complex kinetics with positive cooperative dependence of the reaction rate on [Met] and allosteric regulation by AdoMet [1,2], which activates the enzyme at low and moderate [AdoMet] and inhibits the enzyme at high [AdoMet]. A simplified empirical equation for MATIII reaction rate was developed using the following assumptions: (a) each subunit has one catalytic methionine-binding site and two allosteric sites for AdoMet; (b) methionine binds to MATIII with positive cooperativity; (c) binding of AdoMet to the activating allosteric site activates the enzyme by decreasing the methionine dissociation constant in the catalytic site [1]; (d) binding of AdoMet to the inhibiting allosteric site completely inhibits enzymatic activity of this subunit.

The kinetic scheme for MATIII with two-identical subunits with one catalytic and one allosteric site in each subunit is shown in Figure S1. Assuming that the reversible stages of substrate and effector binding are in equilibrium, then equation S11 describes the corresponding reaction rate:

$$V=\frac{2[E_{0}]\left[\left(k_{f1}+k_{f2}\frac{2[M]}{K_{M}}+k_{f3}\frac{[M]^{2}}{K_{M}^{2}}\right)+\frac{\beta}{[S]}\left(k_{f1}K_{S1}+k_{f2}K_{S2}\frac{2[M]}{K_{M}}+k_{f3}K_{S3}\frac{[M]^{2}}{K_{M}^{2}}\right)\right]}{\left(1+\frac{2[M]}{K_{M}}+\frac{[M]^{2}}{K_{M}^{2}}\right)+\frac{2\beta}{[S]}\left(K_{S1}+K_{S2}\frac{2[M]}{K_{M}}+K_{S3}\frac{[M]^{2}}{K_{M}^{2}}\right)+\frac{1}{[S]^{2}}\left(K_{S1}^{2}+K_{S2}^{2}\frac{2[M]}{K_{M}}+K_{S3}^{2}\frac{[M]^{2}}{K_{M}^{2}}\right)}$$

(Eq.S11)

Here [S], [M], and [E_0_] denote concentrations of substrate, effector, and the total concentration of all enzyme forms, respectively. Constants are designated in Figure S1 legend. Parameter β determines the level of enzyme cooperativity [3]. When β=0, the equation is transformed to the Hill equation for two substrate binding sites, when β=1, the equation represents the Michaelis-Menten dependence on substrate. The consequences of binding of activator or inhibitor to the allosteric site in accordance with Figure S1 are described below.

*Activation of MATIII by AdoMet-*If activator binding to the allosteric site diminishes the substrate dissociation constant without affecting product-forming step: K_S1_=K_S_; K_S2_=ε_1_K_S_; K_S3_=ε_2_K_S_; k_f1_=k_f2_=k_f3_=k_f0_, then Eq.S11 transforms to the following form:

$\begin{matrix} V=A_{0}\frac{1+\frac{\beta K_{S}}{[S]}\theta_{1}}{1+\frac{2\beta K_{S}}{[S]}\theta_{1}+\frac{K_{S}^{2}}{[S]^{2}}\theta_{2}} \\ \theta_{1}=\frac{K_{M}^{2}+2K_{M}[M]\varepsilon_{1}+[M]^{2}\varepsilon_{2}}{\left(K_{M}+[M]\right)^{2}}; \theta_{2}=\frac{K_{M}^{2}+2K_{M}[M]\varepsilon_{1}^{2}+[M]^{2}\varepsilon_{2}^{2}}{\left(K_{M}+[M]\right)^{2}} \end{matrix}$ (Eq.S12)

In case of MATIII, S corresponds to methionine and M corresponds to AdoMet. Equation S12 provides the best fit to the experimental data obtained *in vitro* with purified MATIII [1] when 0≤ε_1_≤0.5 and 0≤ε_2_≤0.08 (Figure S2).

We choose values of ε_1_=0.5 and ε_2_=0, at which the expressions for θ_1_ and θ_2_ are simplified to:

$\theta_{1}=\frac{K_{M}}{K_{M}+[M]}$, $\theta_{2}=\frac{K_{M}^{2}}{\left(K_{M}+[M]\right)^{2}}$ (Eq.S13)

We also introduce an “effective” dissociation constant:

$K^{eff}_{S}=\frac{K_{S}}{1+[M]/K_{M}}$ (Eq.S14)

In the case of MATIII, we obtain the following equation describing the dependence of the enzyme reaction rate on methionine concentration and on AdoMet activation:

$V=A_{0}\frac{1+\frac{\beta K_{S,eff}^{MATIII}}{[Met]}}{1+\frac{2\beta K_{S,eff}^{MATIII}}{[Met]}+\frac{\left(K_{S,eff}^{MATIII}\right)^{2}}{[Met]^{2}}}=A_{0}\frac{[Met]^{2}+\beta K_{S,eff}^{MATIII}[Met]}{[Met]^{2}+2\beta K_{S,eff}^{MATIII}[Met]+\left(K_{S,eff}^{MATIII}\right)^{2}}$ (Eq.S15)

$$K_{S,eff}^{MATIII}=\frac{K_{S0}^{MATIII}}{1+[AdoMet]/K_{A}^{MATIII}}$$

# *Inhibition of MATIII by AdoMet-*If inhibitor binding to the allosteric site completely inhibits enzymatic activity of the subunit: K_S1_=K_S2_=K_S3_=K_S_; k_f1_= k_f0_, k_f2_=1/2·k_f0_, k_f3_=0, then the (Eq.S11) transforms to the following form:

$V=\frac{A_{0}}{1+[M]/K_{M}}\cdot\frac{[S]^{2}+K_{S}\beta[S]}{[S]^{2}+2K_{S}\beta[S]+K_{S}^{2}}$ (Eq.S16)

In case of MATIII we obtain the following equation, which provides the best fit for *in vitro* experimental data [1] describing MATIII inhibition at high AdoMet concentration (Figure S2):

$V=\frac{A_{0}}{1+[AdoMet]/K_{i}^{MATIII}}\cdot\frac{[Met]^{2}+K_{S0}^{MATIII}\beta[Met]}{[Met]^{2}+2K_{S0}^{MATIII}\beta[Met]+K_{S0}^{MATIII2}}$ (Eq.S17)

*Final equation-*The final equation for the reaction rate of MATIII is obtained as a combination of equations S15 and S17:

(Eq.S18)

This equation describes the positive cooperative dependence of the reaction rate on [Met] as well as MATIII activation and inhibition at different AdoMet concentrations. The best description of experimental data showing the dependence of the MATIII reaction rate on [Met] and [AdoMet], obtained *in vitro* with purified enzyme [1] is achieved at parameter values listed in the legend to Figure S2. However, the best description of our experimental results, obtained in suspension of murine hepatocytes is achieved at somewhat different parameter values, listed in Table S1.

**REFERENCES**

1. Sullivan DM, Hoffman JL (1983) Fractionation and kinetic properties of rat liver and kidney methionine adenosyltransferase isozymes. Biochemistry 22: 1636-1641.

2. Cabrero C, Puerta J, Alemany S (1987) Purification and comparison of two forms of S-adenosyl-L-methionine synthetase from rat liver. Eur J Biochem 170: 299-304.

3. Hofmeyr JH, Cornish-Bowden A (1997) The reversible Hill equation: how to incorporate cooperative enzymes into metabolic models. Comput Appl Biosci 13: 377-385.
