# Supplementary material for: An Allosteric Mechanism for Switching between Parallel Tracks in Mammalian Sulfur Metabolism
Source: PLoS Comput Biol. 2008 May 2;4(5):e1000076. doi: 10.1371/journal.pcbi.1000076 (PMC2346559; doi:10.1371/journal.pcbi.1000076)
Supplement: Text S3 — Mechanism of bistability in the model. (0.03 MB DOC) [file pcbi.1000076.s011.doc]

**Text S3. Mechanism of bistability in the model.**

The mechanism of bistability in our model can be described by considering the rates of production and consumption of AdoMet that is a key regulatory intermediate in methionine metabolism. Figure S3 shows the dependence of both rates on [AdoMet] at three different methionine concentrations. We changed MATI activity in the model from 2.2 mmol/h l cells to 1.0 mmol/h l cells when calculated curves in Figures S3 and S4 to make the figures more clear. At low [AdoMet], the rate of AdoMet production decreases with an increase in [AdoMet] due to product inhibition of MATI by AdoMet, and increases at higher [AdoMet] due to activation of MATIII [1]. The rate of AdoMet consumption increases monotonically due to the increase in the rate of functional methylases at low [AdoMet], and due to activation of GNMT at higher [AdoMet]. Intersections between curves represent equivalence in AdoMet production and consumption rates and correspond to steady-state AdoMet concentrations. There is only one intersection at low methionine concentrations corresponding to one stable steady-state under normal physiological conditions (Fig. S3A). An increase in methionine concentration leads to an upward shift of the curve representing the rate of AdoMet production without altering the dependence of AdoMet consumption rate on AdoMet levels. It leads to the appearance of additional intersections and to the simultaneous existence of three steady states (Fig. S3B). The original steady state corresponding to lower [AdoMet] (“low”-state) and the steady state corresponding to highest [AdoMet] (“high”-state) are stable while the intermediate one (point 2 in Fig. S3B) is unstable. Any incidental decrease or an increase in [AdoMet] in this steady state stimulates a transition in metabolism to a lower or higher steady state respectively. So methionine metabolism is a bi-stable system for a certain range of parameters. A further increase in methionine concentration (with a corresponding increase in AdoMet production rate) brings the lower and intermediate steady states closer till they merge and disappear. The only steady state that remains is at high AdoMet concentration (Fig. S3C).

If the methionine concentration increases from low values, the metabolic system remains in the low steady state mode until a threshold methionine concentration is reached. Beyond this value, the lower steady state mode disappears and the system jumps abruptly to the higher steady state. This is illustrated in Fig. S4A, which depicts the dependence of steady state [AdoMet] on methionine levels. When methionine values increase to 84.5 μM, the system jumps to the higher steady state. However, if the system is already in the high steady state mode, it returns to the low mode only when the methionine concentration decreases to 74.7 µM. This difference in the value of a parameter in the forward and backward transition is known as hysteresis and is observed in a number of physical and chemical processes. Figure S4 shows that transitions between low and high steady state modes in the model are associated with corresponding transitions between remethylation and transsulfuration fluxes in methionine metabolism. In the low steady states a significant fraction of methionine is reproduced via remethylation reactions (more than 50% at 50 μM methionine), and this fraction decreases with increasing methionine concentration. When methionine metabolism jumps to the high steady state, the ratio between transsulfuration and remethylation metabolic fluxes increases abruptly more than two times, and methionine is metabolized mainly via transsulfuration pathway in high steady states.

The existence of bistability and hysteresis in the model is a consequence of the kinetic properties of enzymes, viz. MATIII activation by AdoMet, GNMT cooperativity, and the ratio between the activities of MATI and MATIII. It is unknown so far how the ratio between MATI and MATIII activities is controlled in the cells. Our model predicts that the increase in MATI activity at constant MATIII activity leads to a decrease in the area of bistability, which is associated with the negative slope for the dependence of steady-state [AdoMet] on methionine levels (Fig. S5). The model provides the best fit to the experimental results at a value of MATI activity of ~2.2 mmol/h•l cells (Fig. 3) where the bistability area is very small or absent. However, the trigger behavior persists in the model when bistability disappears and occurs because the curves representing the dependencies of AdoMet production and consumption on [AdoMet] have identical slopes in a wide range of [AdoMet] (Fig. S3).

# REFERENCES

1. Sullivan DM, Hoffman JL (1983) Fractionation and kinetic properties of rat liver and kidney methionine adenosyltransferase isozymes. Biochemistry 22: 1636-1641.
